# Structurally Optimized Potent Dual-Targeting NBTI Antibacterials with an Enhanced Bifurcated Halogen-Bonding Propensity

**DOI:** 10.1021/acsmedchemlett.1c00345

**Published:** 2021-08-16

**Authors:** Maja Kokot, Matjaž Weiss, Irena Zdovc, Martina Hrast, Marko Anderluh, Nikola Minovski

**Affiliations:** † Theory Department, Laboratory for Cheminformatics, National Institute of Chemistry, Hajdrihova 19, SI-1001 Ljubljana, Slovenia; ‡ The Chair of Pharmaceutical Chemistry, Faculty of Pharmacy, University of Ljubljana, Aškerčeva cesta 7, SI-1000 Ljubljana, Slovenia; § Veterinary Faculty, Institute of Microbiology and Parasitology, University of Ljubljana, Gerbičeva 60, SI-1000 Ljubljana, Slovenia

**Keywords:** NBTIs, DNA gyrase, Topoisomerase IV, Bifurcated halogen bonds, Dual targeting

## Abstract

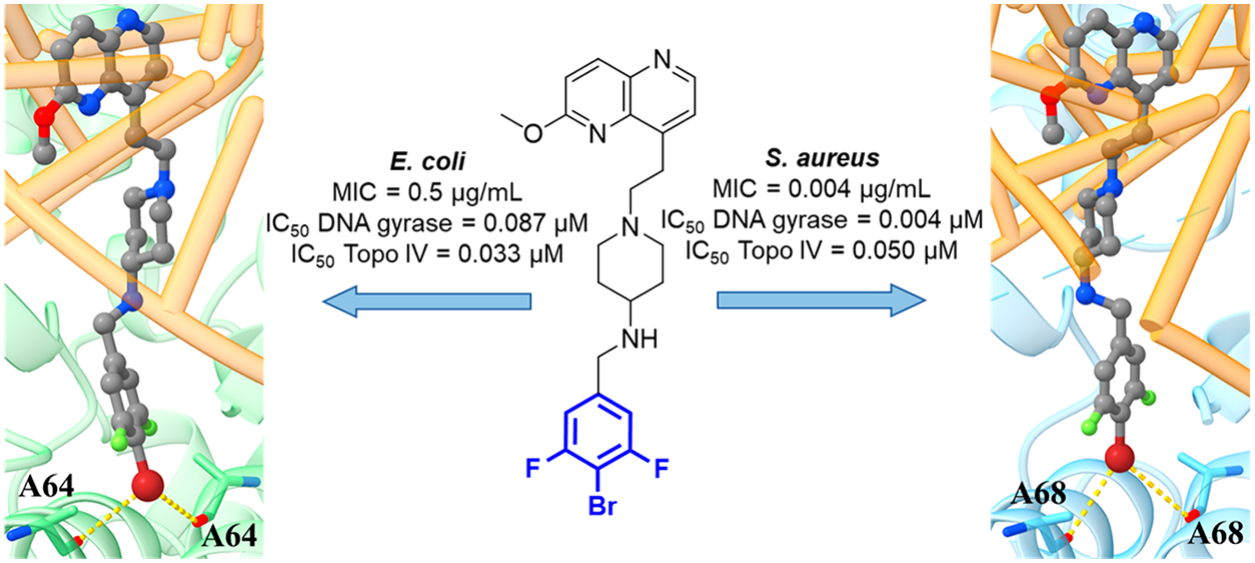

We designed and synthesized an optimized library of novel bacterial topoisomerase inhibitors with *p*-halogenated phenyl right-hand side fragments and significantly enhanced and balanced dual-targeted DNA gyrase and topoisomerase IV activities of *Staphylococcus aureus* and *Escherichia coli*. By increasing the electron-withdrawing properties of the *p*-halogenated phenyl right-hand side fragment and maintaining a similar lipophilicity and size, an increased potency was achieved, indicating that the antibacterial activities of this series of novel bacterial topoisomerase inhibitors against all target enzymes are determined by halogen-bonding rather than van der Waals interactions. They show nanomolar enzyme inhibitory and whole-cell antibacterial activities against *S. aureus* and methicillin-resistant *S. aureus* (MRSA) strains. However, due to the relatively high substrate specificity for the bacterial efflux pumps, they tend to be less potent against *E. coli* and other Gram-negative pathogens.

Bacterial type II topoisomerases are well-validated targets for antibacterial chemotherapy, including DNA gyrase and its paralogous form topoisomerase IV (topoIV). These molecular nanomachines are involved in maintaining the correct topological state of the DNA in bacteria and consequently in regulating vital bacterial processes such as cell replication, gene transcription, and genetic recombination.^1^ DNA gyrase is responsible for the introduction of negative supercoils into the DNA molecule, and topoIV is responsible for the DNA decatenation activity. Consequently, inhibiting the function of either or both of these enzymes leads to perturbations in the native spatial DNA topology, which results in bacterial cell death.^2−4^

From the plethora of antibacterials that target bacterial topoisomerases, 6-fluoroquinolones can be regarded as one most widely used intercalating agents in antibacterial chemotherapy.^3^ Despite their long-standing value in clinical practice, they now show a decreased potency, mainly as a consequence of the increased quinolone-based “acquired resistance” in bacteria.^5^ Still, some quinolones continue to be approved for clinical use. In 2017, the U.S. Food and Drug Administration (FDA) approved delafloxacin for acute bacterial skin infections.^6^ Delafloxacin has a chemically distinct structure compared to the currently marketed fluoroquinolones; the absence of a protonatable substituent confers a weakly acidic character to the molecule that enhances its antibacterial activity in acidic environments such as one that occurs in *Staphylococcus aureus* infections.^7,8^ Recently, a new class of promising antibacterials known as “novel bacterial topoisomerase inhibitors” (NBTIs) has been discovered.^9−12^ They differ from 6-fluoroquinolones not only in their structure, but also in their binding mode and consequently their mechanism of action. Compared to 6-fluoroquinolones, NBTIs bind to a different, but vicinal and nonoverlapping binding site in bacterial topoisomerases, thus avoiding cross-resistance with 6-fluoroquinolones. Furthermore, while the mechanism of the bacterial topoisomerase inhibition of 6-fluoroquinolones is based on the stabilization of double-strand DNA breaks, NBTIs stabilize single-strand breaks.^9,13,14^ The most advanced NBTI is gepotidacin, which is currently in the third phase of clinical trials for the treatment of uncomplicated urogenital gonorrhea.^15^ It is notably effective for infections caused by Gram-negative bacteria. However, it shows weaker antibacterial activity against Gram-positive bacteria (phase II clinical trials of gepotidacin for the treatment of acute bacterial skin infections), mainly as a consequence of some point mutations (GyrA D83N and ParC V67A). In particular, the lack of activity of gepotidacin against *S. aureus* topoIV might be a pivotal reason for the emergence of resistant strains.^16^

Structurally, DNA gyrase and topoIV are heterotetrameric enzymes, and their subunits share a high structural similarity in the binding site of NBTIs.^17^ DNA gyrase is comprised of two GyrA subunits and two GyrB subunits (i.e., A2B2), while topoIV is comprised of two ParC subunits and two ParE subunits (i.e., C2E2). This structural similarity allows NBTIs to effectively inhibit both enzymes. Thus, dual targeting should lead to a higher antibacterial efficiency and a lower effective bacterial mutation frequency (i.e., nonspontaneous resistance), with the consequent lower risk for the development of bacterial resistance compared to that of single-target inhibition.^18−20^

Novel bacterial topoisomerase inhibitors have three key parts: a heteroaromatic “left-hand side”, which can intercalate between the central DNA base pairs; a bicyclic or monocyclic heteroaromatic “right-hand side” (RHS), which can bind into the deep noncatalytic hydrophobic binding pockets formed by GyrA of DNA gyrase and ParC of topoIV; and their connecting linker moiety.^17,21^ A basic nitrogen on the linker is required to establish an ionic interaction with Asp83 of the *Staphylococcus aureus* GyrA, which has been shown to be particularly important for NBTIs binding and affinity.^9,11,21,22^

Some of the dual-targeting NBTIs have shown inhibition of both DNA gyrase and topoIV in the same bacterial strain, although their inhibitory potencies are not always balanced.^22−25^ To achieve effective dual targeting that can lead to synergistic antibacterial effects, NBTIs need to be optimized toward the balanced inhibition of both of these enzymes (Figure 1). This can be achieved by carefully examining the DNA gyrase and topoIV binding sites and taking into account the inherent differences in the binding site topology when designing and optimizing new NBTIs.

**Figure 1:**
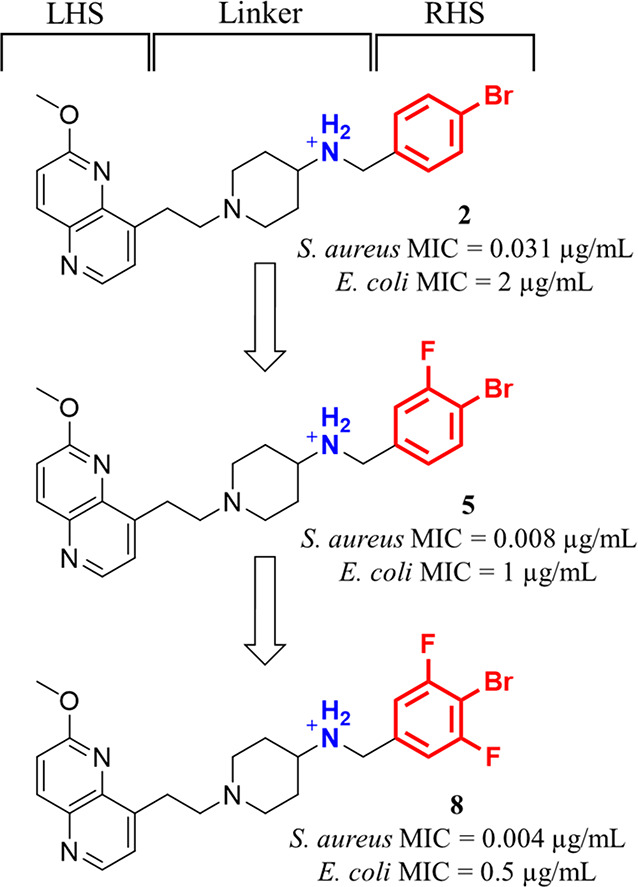
Structural optimization strategy for the improved antibacterial activities of NBTIs, from **2** to **5** to **8**. Here, the *p*-halogenated RHS fragments are enhanced in terms of their bifurcated halogen-bonding propensity for targeting bacterial type II topoisomerases. Red bold shows the optimized RHS moieties, and blue bold shows the protonated basic amine that binds to Asp83 of *S. aureus* GyrA.

The crystal structure of *S. aureus* DNA gyrase in complex with the low nanomolar efficacy NBTI GSK299423 and DNA revealed that GSK299423 establishes atypical hydrogen bonds with the Ala68 residues of both the GyrA subunits (Figure S1a).^9^ Based on this observation, we recently designed a series of NBTIs with *p*-halogenated phenyl RHS fragments.^13^ We demonstrated that the halogen atom at the *para*-position on the phenyl RHS fragment (e.g., Cl, Br, or I) can indeed establish strong symmetrical bifurcated halogen bonds with the backbone carbonyl oxygens of these Ala68 residues, which are thus responsible for the high antibacterial potencies. This was verified in our recently solved crystal structure of *S. aureus* DNA gyrase in complex with an NBTI (**1**) that included *p*-chloro phenyl on the RHS fragment (PDB ID 6Z1A, Figure S1b).^13^

However, although these compounds showed very potent inhibition of DNA gyrase activity, they did not show the balanced inhibition of both DNA gyrase and topoIV within the same bacterial strain. This was particularly evident for compound **1**, the inhibitory potency of which differs between *S. aureus* DNA gyrase and topoIV by almost three orders of magnitude (Table 1). Therefore, with the aim to achieve enhanced NBTI binding to both these enzyme within a single bacterial strain and thus to provide more potent antibacterial activity, we focused on the key structural similarities between DNA gyrase and topoIV.

**Table 1:**
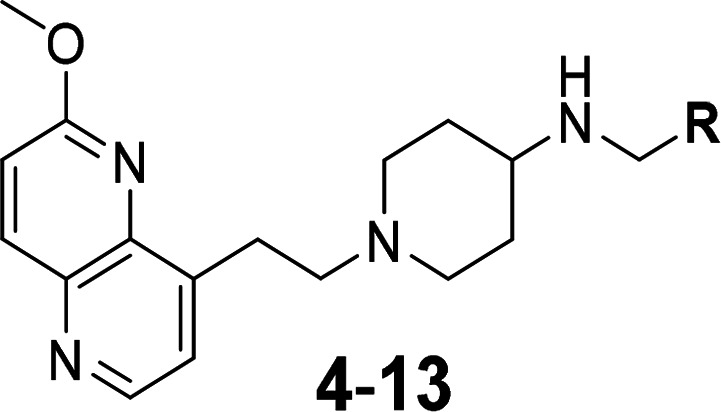

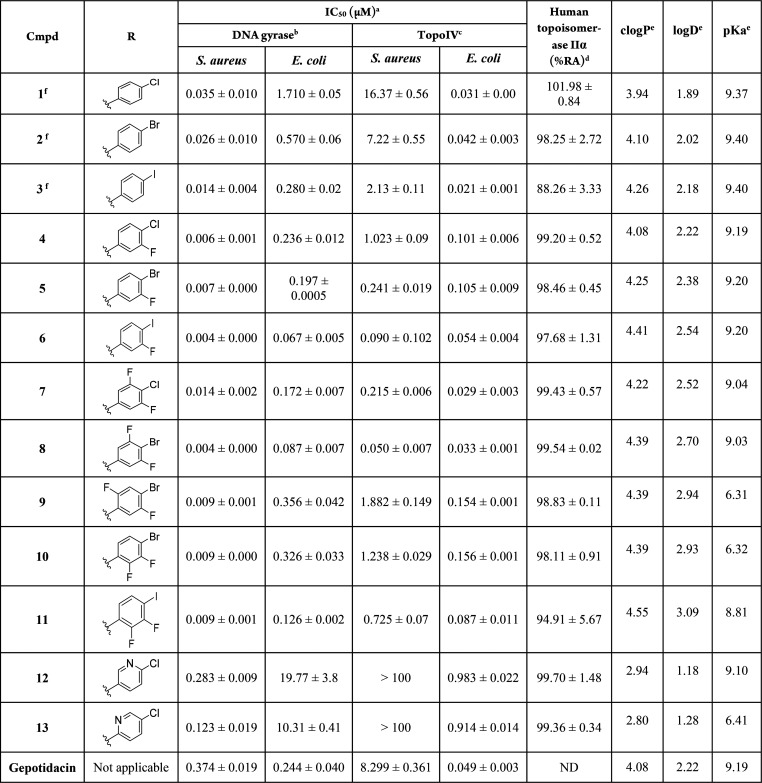
*S. aureus* and *E. coli* DNA Gyrase, TopoIV, and Human Topoisomerase IIα Inhibitory Activity and Physico-Chemical Properties of the Optimized Series of NBTIs

a Means of two independent measurements ± SD.

b DNA gyrase supercoiling inhibition assay.

c TopoIV decatenation inhibition assay.

d Means ± SD of the residual activity (%) at a 10 μM compound concentration from two independent experiments.

e clogP, logD, and p*K*_a_ were calculated with MarvinSketch ver. 20.17. logD and p*K*_a_ values are calculated at pH 7.4; ND, not determined.

f Ref (31).

As the *S. aureus* and *Escherichia coli* DNA gyrase and topoIV share the same pair of alanine backbone carbonyls (Figure 2), a more potent and balanced inhibitory activity might be obtained by increasing the strength of the bifurcated halogen-bonding. This can be achieved by introducing electron-withdrawing groups at the *ortho*- and *meta*-positions of the *p*-halogenated phenyl RHS fragments in these NBTIs (Figure 1).^26^ In this way, we designed a small series of structurally optimized NBTIs with differently fluorinated *p*-halogenated phenyl RHS fragments (Table 1). The binding modes of these NBTIs were then predicted through flexible molecular docking calculations for the AMK-12 binding pocket of the *S. aureus* DNA gyrase (PDB ID 6Z1A)^13^ (i.e., the gepotidacin binding site of the cryo-electron microscopy structure of *E. coli* DNA gyrase; PDB ID 6RKS)^27^ (see the Supporting Information). Moreover, to better define the dual targeting, the binding mode of this optimized NBTI series was also predicted through flexible molecular docking calculations using our in-house-constructed *S. aureus* and *E. coli* topoIV protein homology models.

**Figure 2:**
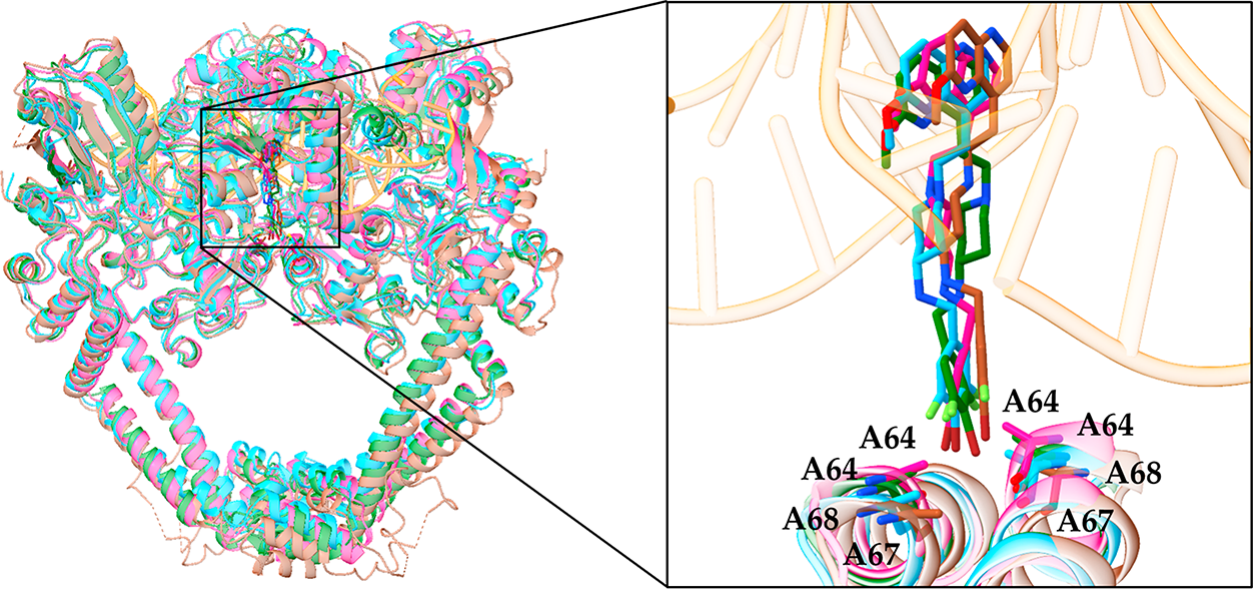
Structural superpositioning of the docked poses of compound **8** for all four enzymes: *S. aureus* DNA gyrase (blue, PDB ID 6Z1A),^13^*E. coli* DNA gyrase (brown, PDB ID 6RKS),^27^ the *S. aureus* topoIV homology model based on the *Streptococcus pneumoniae* topoIV structure as a template (pink, PDB ID 3RAF),^32^ and the *E. coli* topoIV homology model based on the *S. pneumoniae* topoIV structure as a template (green, PDB ID 3KSA).^33^ Enzymes are shown as ribbons, compound **8** is shown as sticks, Ala residues are shown as sticks that are colored by an element, and DNA is shown as orange. For clarity, the docked poses of compound **8** were inserted artificially and colored as their corresponding target enzymes.

Comparisons were then made among the available experimental structural data and our homology models for these four target enzymes (i.e., DNA gyrase and topoIV of *S. aureus* and *E. coli*). These allowed the identification of the crucial variations in the amino acids that delineated the NBTI binding pockets for DNA gyrase and topoIV from both of the bacteria. The key amino acid differences for DNA gyrase were Met75 in *S. aureus* versus Ile74 in *E. coli*; for topoIV, they were Ile71 in *S. aureus* versus Leu71 in *E. coli* (Figure S3).^17,27^

These isoleucines in the *E. coli* DNA gyrase and *S. aureus* topo IV might show steric hindrance for the binding of NBTIs due to spatial changes in the binding pockets.^17^ Moreover, all four of these enzymes are comprised of symmetrically oriented alanine residues in the α3-helices of the GyrA/ParC subunits, which were previously identified as key attachment points for the bifurcated halogen bonds of NBTIs that contain *p*-halogenated RHS fragments (Figure 2).^13^ Nevertheless, the docking results show that all four of the bacterial enzymes can accommodate smaller and mainly monocyclic RHS fragments, such as the proposed *p*-halogenated phenyls.

On this basis, and with the aim of boosting the strength of the halogen bonds (as previously proposed),^28^ we introduced fluorine substituents at the *ortho*- and *meta*-positions of the *p*-halogenated phenyl RHS fragments in these NBTIs. The fluorine substituent was selected because of two important properties: its size is similar to that of a hydrogen atom, making it one of the smallest substituents to appropriately fit the sterically limited RHS binding sites, and it is a strong electron-withdrawing group that can increase the σ-hole of the adjacent *p*-substituted halogen, thereby intensifying the halogen-bonding propensity and strength by increasing not only the σ-hole size but also its positive potential.^29,30^ Considering that the size of the σ-hole is particularly important in allowing the optimal geometry of the bifurcated halogen-bonding (increase in the allowed Θ_1_ angle), we opted to intensify the propensity and strength by introducing the fluorine substituents on the aromatic ring to increase the size of the σ-hole.^13^ The synthesis of these NBTIs is given in detail in the Supporting Information, resulting in the focused library of an optimized series of NBTIs that is presented in Table 1. These NBTIs showed potent activities against the *S. aureus* DNA gyrase and *E. coli* topoIV that were in the low nanomolar range, although they were relatively less potent for the inhibition of the *E. coli* DNA gyrase and *S. aureus* topoIV.

These data are in agreement with our previous findings that the potencies of this series of NBTIs that include the *p*-halogenated phenyl RHS fragments increase according to the increasing size and polarizability of the halogen atoms at the *para*-position on the phenyl ring^13,31^ as well as their σ-hole size.^26,34^ As demonstrated by the data in Table 1, significantly improved inhibitory potencies were obtained for the optimized series of compounds **4**–**11** compared to the previously tested compounds **1**–**3**^31^ for all targeted enzymes, except for *E. coli* topoIV. With the exceptions of **4** and **5**, where the inhibitory potencies were similar, there was a particular improvement in the inhibitory potency of **6** against all four enzymes. Moreover, upon the enrichment of the *p*-halogenated phenyl RHS fragments by the incorporation of an additional fluorine, the σ-hole size of the RHS fragment *p*-halogen atom increased even further,^26,34^ which additionally enhanced the strength of the halogen bonds. Monofluorinated analogs **4**–**6** are generally more potent compared to the previously tested nonfluorinated **1**–**3**.^31^ Accordingly, the difluorinated compounds **8** to **10** showed even higher inhibitory potencies, where **8** contained the symmetrical *m*-difluoro substitution and was the most potent.

Comparisons of the on-target potencies of **8**–**10** show that the increased lipophilicity was not the main driving force for potency here; instead, it was the bifurcated halogen-bonding. Namely, **8**–**10** had very similar logP values and would thus be expected to be similarly potent if the lipophilicity was the pivotal physicochemical property governing their potency (Table 1). Instead, the highest potency is observed for **8**, which has the highest electron-withdrawing effect, and thus the largest σ-hole size due to the optimal fluorine positions. The molecular docking calculations predicted that **8** could establish bifurcated halogen-bonding interactions with the backbone carbonyl oxygens of Ala68 in the *S. aureus* GyrA and Ala64 in *E. coli* ParC (Figure 3).

**Figure 3:**
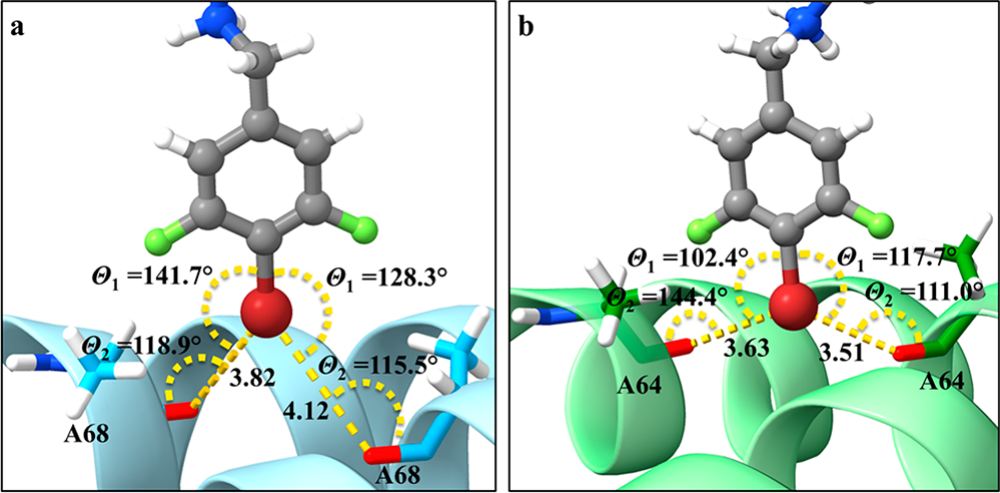
Prediction of bifurcated halogen bonds for **8** (gray ball and sticks) with (a) the backbone carbonyl oxygens of Ala68 (cyan sticks) from both GyrA subunits of the *S. aureus* DNA gyrase (light blue ribbon, PDB ID 6Z1A)^13^ and (b) Ala64 (cyan sticks) from both ParC subunits of the *E. coli* topoIV homology model (green ribbon). The halogen-bonding interactions are shown as yellow dots.

As well as this apparent increase in the strength of the halogen bonds, there are three further potential reasons why the highest potency is visible for **8**: (i) the symmetry of the binding site, (ii) the larger RHS fragment volume and consequently the additional van der Waals interactions of the fluorinated analogs, and (iii) the protonation of the secondary amine of the linker relative to the position of the electron-withdrawing fluorine. Put differently, the symmetry of the binding site provides an appropriate accommodation for the symmetric RHS fragments. Analogously, the symmetrically difluorinated phenyl RHS fragments appear to be better accommodated within the GyrA/ParC binding sites relative to the monosubstituted RHS fragments.

Comparisons of the *S. aureus* and *E. coli* DNA gyrase and topoIV potencies of **8** (Figure S4) indicate that it has an equilibrated activity on all selected targets that could lead to a synergy of its antibacterial activities (Table 2). However, Nayar et al. reported that the DNA gyrase inhibitor NBTI 5463 that showed balanced on-target activities did not lead to synergy against *E. coli*, which contradicts the proposed synergistic effects of **8**.^35^ In our case, we believe that the lack of synergy may be explained with the different importance of each enzyme in provoking antibacterial activity. The latter can be scrutinized by correlating the antibacterial effects of structurally related compounds **2** and **8** on *S. aureus* and *E. coli* with the inhibitory activity of both compounds on the DNA gyrase and topoIV of both bacteria. Namely, there is a more than two orders of magnitude difference between the potencies of **2** and **8** on *S. aureus* topoIV, while the difference in potency on DNA gyrase is less than one order of magnitude. If topoIV would be an important target, the potency difference should be reflected in a much lower MIC values for **8** on *S. aureus*; however, this is not the case as the difference in MICs between **2** and **8** is less than one order of magnitude. This is almost exactly the difference in the potency on DNA gyrase, showing that for *S. aureus* DNA gyrase is the primary target. In the case of *E. coli*, the compounds **2** and **8** inhibit topoIV even more potently than DNA gyrase, and topoIV inhibition is almost equipotent. Should topoIV be an important target, this would result in almost the same MICs. Yet, there is a significant difference between the MIC values of **2** and **8** on wild-type *E. coli*, and the difference in MICs roughly corresponds to the difference in the potency on the isolated DNA gyrase. Taken together, this comparison strongly suggests that the antibacterial effect is predominantly the consequence of DNA gyrase inhibition in both bacteria. A similar conclusion can also be drawn for compounds **7** and **8** when comparing their antibacterial potencies with inhibition data on isolated enzymes. This raises the question of whether the synergistic effect might be obtained through the dual targeting of DNA gyrase and topoIV. Nayar et al. indicated that just a single target mutation (either in GyrA or ParC) did not markedly affect the antibacterial activity of NBTI 5463 against *E. coli*. Accordingly, it can be concluded that even if a synergistic effect is not achieved, dual targeting is still a plausible way to avoid resistance due to single-target mutations.

**Table 2:** Antimicrobial Susceptibility of the Optimized NBTIs against a Panel of Gram-Positive and Gram-Negative Bacterial Pathogens

	MIC (μg/mL)													
compound	1^a^	2^a^	3^a^	4	5	6	7	8	9	10	11	12	13	Gepo
*S. aureus* (ATCC 29213)	0.125	0.031	0.008	0.031	0.008	0.008	0.008	0.004	0.008	0.031	0.008	1	0.125	0.125
*E. coli* (ATCC 25922)	4	2	2	2	1	0.5	0.5	0.5	1	4	2	32	32	1
*E. coli* D22^b^	2	0.125	0.125	0.25	0.062	0.062	0.031	0.016	0.25	0.5	0.125	4	16	0.125
*E. coli* N43^c^ (CGSC no. 5583)	0.5	0.125	0.078	0.062	0.031	0.016	0.062	0.008	0.062	0.125	0.031	8	8	0.016
MRSA (QA-11.7)^d^	0.5	0.062	0.008	0.031	0.016	0.016	0.031	0.016	0.016	0.062	0.031	1	0.25	0.062
MRSA (QA-12.1)^e^	ND	ND	ND	0.031	0.031	0.016	0.031	0.016	0.016	0.062	0.062	2	0.25	0.125
*Klebsiella pneumoniae*	ND	ND	ND	32	16	8	8	8	64	64	32	>128	>128	8
*Salmonella alachua* RDK 030c (QA-1482/04)	32	16	8	8	4	2	2	1	16	16	8	>128	>128	4
*Pseudomonas aeruginosa* RDK 184 (DSM 939)	>128	128	32	64	32	16	16	8	128	>128	64	>128	>128	8
*Streptococcus agalactiae* RDK 047 (QA-990/02)	1	0.5	0.125	1	2	0.25	0.5	0.25	0.25	2	0.5	64	8	ND
*Enterococcus faecalis* DRK 057 (ATCC 29212)	1	1	1	2	0.5	0.25	0.5	0.5	0.5	2	0.5	32	4	ND

a Ref (31). ATCC, American Type Culture Collection; CGSC, Coli Genetic Stock Centre; QA, quality assurance; DSM, German Collection of Microorganisms and Cell Cultures; ND, not determined; Gepo, gepotidacin.

b With a mutation in the *lpxC* gene that increases membrane permeability.

c With the *AcrA* knockout (cell membrane efflux pump).

d Resistant to cefoxitin, ciprofloxacin, clindamycin, erythromycin, tetracycline, thiamulin, and trimethoprim.

e Resistant to cefoxitin, gentamicin, kanamycin, rifampicin, streptomycin, sulfamethoxazole, and tetracycline.

The effects of basicity and the p*K*_a_ of the secondary amine on the linker of these NBTIs influences their ionization and their propensity to form an ionic interaction with the aspartic acid residue (Table 1). Thus, when an electron-withdrawing fluorine is introduced near the basic nitrogen, the basicity of the amine is reduced due to the σ-inductive effect of the fluorine atom.^36^ This can be seen for **8**–**10**, where the decrease in the basicity of the secondary amine is stronger when the fluorine atom on the phenyl RHS fragment is in the *ortho*-position compared to that for the *meta*-position. The p*K*_a_ values for **9** and **10** (where the fluorine atom is spatially closer to the amine) are 6.31 and 6.32, respectively, indicating that the amine is mainly in an unprotonated form at a physiological pH and is thus preventing ionic interactions with the GyrA Asp83 of DNA gyrase and the ParC Asp79 of topoIV. The p*K*_a_ of the secondary amine in **8** is 9.30, whereby the majority of **8** is in the protonated form at a physiological pH. The protonated form interacts with one GyrA Asp83 or one ParC Asp79 through an ionic interaction that is crucial for the antibacterial activity.^17^

To show that the nitrogen in the pyridine does not increase the σ-hole size (unlike the substitution with fluorine), we introduced pyridine instead of phenyl as the RHS core aromatic substituent. Both compounds with pyridine RHS **12** and **13** show only about one tenth the inhibitory potency of **1**, as was similarly observed by Li et al.^24^ and Singh et al.^37^ The derivative with the nitrogen in the *ortho*-position (**13**) showed slightly better results compared to that with the nitrogen in the *meta*-position (**12**). However, the drop in potency compared to that of phenyl analog **1** shows that the mesomeric effect of the ring nitrogen does not influence the σ-hole size of the *p*-halogen, unlike the inductive effect for the fluorine substituent at the same position.

Despite the very balanced on-target inhibitory activities against both DNA gyrase and topoIV from *S. aureus* and *E. coli* (Table 1), these optimized NBTIs are generally more effective against Gram-positive bacteria (e.g., *S. aureus* and MRSA strains) than against *E. coli* as a representative Gram-negative bacterium (see the Supporting Information, Figure S5). The potency was improved for the *AcrA* knockout strain of *E. coli* N43 (the strain lacking the cell membrane efflux pump) and also to a lesser extent for the strain with a mutation in the *lpxC* gene (which increases the membrane permeability) of *E. coli* D22 (see the Supporting Information, Figure S6) compared to that of wild-type *E. coli*. This shows that our compounds have two shortcomings related to their inhibition of the growth of Gram-negative bacteria. First, their membrane permeability is suboptimal for crossing the membranes of Gram-negative pathogens and, even more importantly, they are efficiently pumped-out by the *E. coli* efflux pumps. This results in reduced overall cellular activities against *E. coli* and most probably against other Gram-negative bacteria (e.g., *Klebsiella pneumoniae*, *Salmonella alachua*, and *Pseudomonas aeruginosa*), as summarized in Table 2.

Compounds **6**–**8** are significantly more potent against *E. coli* topoIV than the other compounds here (Table 1), and their antibacterial potencies against *E. coli* are comparable to those of some drugs used as antibacterials (e.g., tetracycline). While this is already a notable result, their on-target potencies indicate the conclusion that their whole-cell potency might be further improved by the co-application of efflux pump inhibitors. We plan to evaluate this possibility in future studies.

All these NBTIs show good selectivities for bacterial topoisomerases compared to the orthologous human topoisomerase IIα enzyme (Table 1). Their in vitro safety profiles were determined according to cytotoxicity assessments on the metabolic activities of two specific cell lines: human umbilical vein endothelial cells (HUVECs) and HepG2 liver cancer cells (Table 3). According to their IC_50_ values, compounds **4**–**11** showed weaker effects by up to three orders of magnitude on human cells compared to those for the Gram-positive bacteria (MICs; Supporting Information, Table S3).

**Table 3:** Cytotoxicity Data for Human HUVECs and HepG2 Cells

	IC_50_ (μM)^a^	
compound	HUVECs	HepG2 cells
**4**	31.90 ± 6.56	12.85 ± 1.09
**5**	22.45 ± 5.18	10.78 ± 0.63
**6**	18.47 ± 1.13	9.78 ± 1.33
**7**	29.72 ± 9.35	13.04 ± 1.58
**8**	28.53 ± 4.56	12.44 ± 1.84
**9**	32.85 ± 6.51	14.81 ± 2.64
**10**	29.02 ± 4.95	12.19 ± 2.32
**11**	34.50 ± 3.10	13.28 ± 1.66
**12**	% RA = 70 ± 1^b^	% RA = 52 ± 1^b^
**13**	% RA = 90 ± 2^b^	%RA = 31 ± 3^b^

a Means of three independent measurements ± SD.

b Means ± SD for the residual cell viability at a 50 μM compound concentration from two independent experiments.

The data in Table 3 for **12** and **13** are presented as the residual metabolic activities (%) of cells treated with 50 μM NBTIs from two independent experiments, each of which was performed in triplicate. As demonstrated, **12** and **13** show only partial or little cytotoxicity at 50 μM, suggesting that the potencies against *S. aureus* and *E. coli* do not overlap with the cytotoxicities. Therefore, the more potent compounds against these bacteria should have better safety profiles in terms of effects on human cells. Of note, an important NBTI class-related effect is a tendency to inhibit hERG potassium channels.^38^ Accordingly, to further assess the toxicity profiles of these compounds, it is necessary to evaluate their hERG cardiotoxic potential, which we plan to do in the future.

In summary, we have reported here on the design, synthesis, and biological evaluation of an optimized series of highly potent NBTIs with *p*-halogenated phenyl RHS fragments for the dual targeting of DNA gyrase and topoIV activities. By introducing various fluoro substitutions to the RHS fragments, a good enhancement of the bifurcated halogen-bonding propensity and strength was achieved between the *p*-halogenated phenyl RHS fragments and the Ala68 and Ala64 carbonyl oxygens in the GyrA and ParC hydrophobic binding pockets. These optimized NBTIs showed highly improved enzyme inhibitory potencies that exceed those of previously published NBTIs.

An important conclusion from this series of NBTIs is that, along with the structural data from the crystal structure,^10^ they offer additional evidence for the existence and importance of this halogen-bonding. Namely, by increasing the size of the halogen, the van der Waals contact area can be increased such that just the size and lipophilicity can guide the NBTI potency. However, with this new series we show that their potencies are amplified by increasing the electron-withdrawing properties of the phenyl substituents while retaining the same lipophilicity and approximately the same size. This indicates that halogen-bonding rather than van der Waals bonding guides the observed potencies. Moreover, these compounds showed relatively well-balanced inhibitory activities against both *S. aureus* and *E. coli* due to the appropriate steric positioning of the optimized RHS fragments in the enzyme binding sites. For Gram-positive bacteria (e.g., *S. aureus* and various MRSA clinical isolates), it is evident that our NBTIs have satisfied the aims of this study, as exemplified by their high antibacterial potencies. However, they show relatively weaker potencies against *E. coli* and other Gram-negative bacteria. This appears to be because they are substrates of the bacterial efflux pumps, as demonstrated by the significant improvements in MICs for the *AcrA* (efflux pump) knockout of *E. coli* N43. Among these NBTIs, **8** shows the highest enzyme inhibition and whole-cell potency, making it a promising hit for further hit-to-lead optimization toward achieving optimized NBTIs with the desired potencies and safety profiles.

## Supporting Information Available

Details and data for homology modeling, molecular docking, synthesis and characterization of the compounds; NMR spectra; and biological evaluation data (i.e., assay details for inhibitory activities, antimicrobial testing, and cytotoxicity) (PDF)

## Abbreviations

NBTI: novel bacterial topoisomerase inhibitors
MRSA: methicillin-resistant *S. aureus* (MRSA)
topoIV: topoisomerase IV
6-FQs: 6-fluoroquinolones
ATCC: American Type Culture Collection
CGSC: Coli Genetic Stock Center
QA: quality assurance
DSM: German Collection of Microorganisms and Cell Cultures.
