# Supplementary material for: Structurally Optimized Potent Dual-Targeting NBTI Antibacterials with an Enhanced Bifurcated Halogen-Bonding Propensity
Source: ACS Med Chem Lett. 2021 Aug 16;12(9):1478–85. doi: 10.1021/acsmedchemlett.1c00345 (PMC8436411; doi:10.1021/acsmedchemlett.1c00345)
Supplement: Supplementary file 1 — ml1c00345_si_001.pdf [file ml1c00345_si_001.pdf]

Supporting Information

Structurally optimized potent dual-targeting NBTI antibacterials with enhanced bifurcated halogen-bonding propensity

Maja Kokot[a, b], Matjaž Weiss[b], Irena Zdovc[c], Martina Hrast[b], Marko Anderluh*[b], Nikola Minovski*[a]

*Corresponding authors. Tel. +386 1 4760 383, e-mail: nikola.minovski@ki.si, Tel. +386 1 4769 639, e-mail: marko.anderluh@ffa.uni-lj.si

[a] Theory Department, Laboratory for Cheminformatics, National Institute of Chemistry, Hajdrihova 19, SI-1001 Ljubljana (Slovenia), [b] The Chair of Pharmaceutical Chemistry, Faculty of Pharmacy, University of Ljubljana, Aškerčeva cesta 7, SI-1000 Ljubljana (Slovenia), [c] Institute of Microbiology and Parasitology, Veterinary Faculty, University of Ljubljana, Gerbičeva 60, SI-1000 Ljubljana (Slovenia).

Table of Contents

Design and optimization strategy 3
Homology modeling 3
Molecular docking calculations 3
General Chemical Methods 5
Deprotection of NH_2: BOC protect group 6
General procedure: Reductive amination 6
Synthetic processes of individual compounds 7
NMR spectra 14
Biological evaluation 24
Determination of DNA gyrase and topoisomerase IV inhibitory activities 24
Antimicrobial testing 24
Cytotoxycity (Metabolic activity assay) 25
Correlation of inhibitory and antibacterial activity 26
References 27

Design and optimization strategy

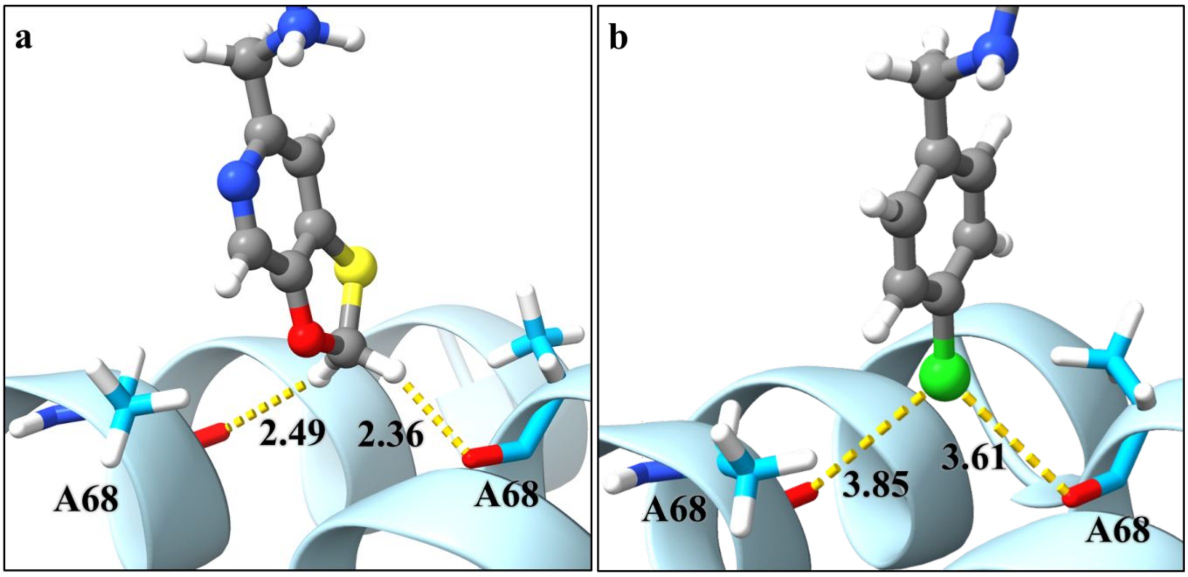

Figure S1: The crystal structures of *Staphylococcus aureus* DNA gyrase enzyme with bound different NBTI ligands: **a)** GSK299423 that establishes atypical hydrogen bonds with the backbone carbonyl oxygens of Ala68 residues from both GyrA subunits (PDB ID: 2XCS), and **b)** AMK-12 ligand that forms bifurcated halogen-bonding interactions with the backbone carbonyl oxygens of the same Ala68 GyrA residues (PDB ID: 6Z1A). Enzyme is shown in cartoon presentation with important amino acid residues in sticks, while NBTIs (sticks and balls representation) are coloured by element.

Homology modeling

Due to the unavailability of any experimental structural information related to topoisomerase IV (topo IV) enzyme originating from *Staphylococcus aureus* and *Escherichia coli*, topo IV protein homology models were constructed utilizing SWISS-MODEL protein homology modeling server.[1] The primary amino acid sequences (publicly available on the UniProt repository) associated to topo IV ParC and ParE subunits for each bacterial organism (*S. aureus* parC: Q2FYS4, parE: Q2FYS5, i.e., *E. coli* parC: P0AFI2, parE: P20083), separately were used as target sequences, while PDB ID: 3RAF and 3KSA were selected as template structures for topo IV homology models constructions. The selection of suitable template structures was done considering the following values as obtained by the SWISS-MODEL: amino acid coverage, GMQE score (Global Model Quality Estimation is a number between 0 and 1 that combines properties from the target–template alignment and the template structure. Higher number indicates higher reliability.), identity, and resolution (Table S1). Once topo IV homology models were assembled, they were additionally visually checked relative to the template structures used.

Table S1: Parameters for suitable template structures selection

	S. aureus	*E. coli*
ParC (protein ID from Uniprot)	Q2FYS4	P0AFI2
ParE (protein ID from Uniprot)	Q2FYS5	P20083
Template protein (PDB ID)	3RAF[2]	3KSA[3]
GMQE	0.45	0.41
Identity	68.3	43.0
Resolution	3.2	3.3

Molecular docking calculations

To account for dual-targeting, all newly designed NBTIs were docked utilizing GOLD docking suite in a flexible fashion[4] employing *S. aureus* DNA gyrase crystal structure complex with AMK-12 ligand (PDB ID: 6Z1A)[5] as well as the *E. coli* DNA gyrase cryoEM structure in complex with gepotidacin (PDB ID: 6RKS)[6]. The experimental coordinates of the natively present NBTI ligands AMK-12 and gepotidacin in *S. aureus* and *E. coli* DNA gyrase, respectively, were used for defining the binding site (cavity radius of 15.5 Å) in both enzymes. Each compound was docked up to 10 times into the defined binding site using the same settings and parameters of the GOLD genetic algorithm (population size = 100, selection pressure = 1.1, number of operations = 100,000, number of islands = 5, niche size = 2, migrate = 10, mutate = 95, cross-over = 95). The amino acid residues Met75, Asp83, and Met121 in *S. aureus* GyrA and Ile74, Asp82, and Met120 in *E. coli* GyrA were considered flexible during the docking calculations. The molecular docking protocol was initially validated by re-docking of the natively present AMK-12, i.e., gepotidacin ligand three times within their corresponding binding site (Figure S1). As a decisive criteria for the quality of all performed structure-based settings,[6] the heavy-atoms root-mean-square deviation (RMSD $\leq$ 2.0Å) values between each calculated docking solution and AMK-12, i.e., gepotidacin ligand conformation were calculated (Table S2).

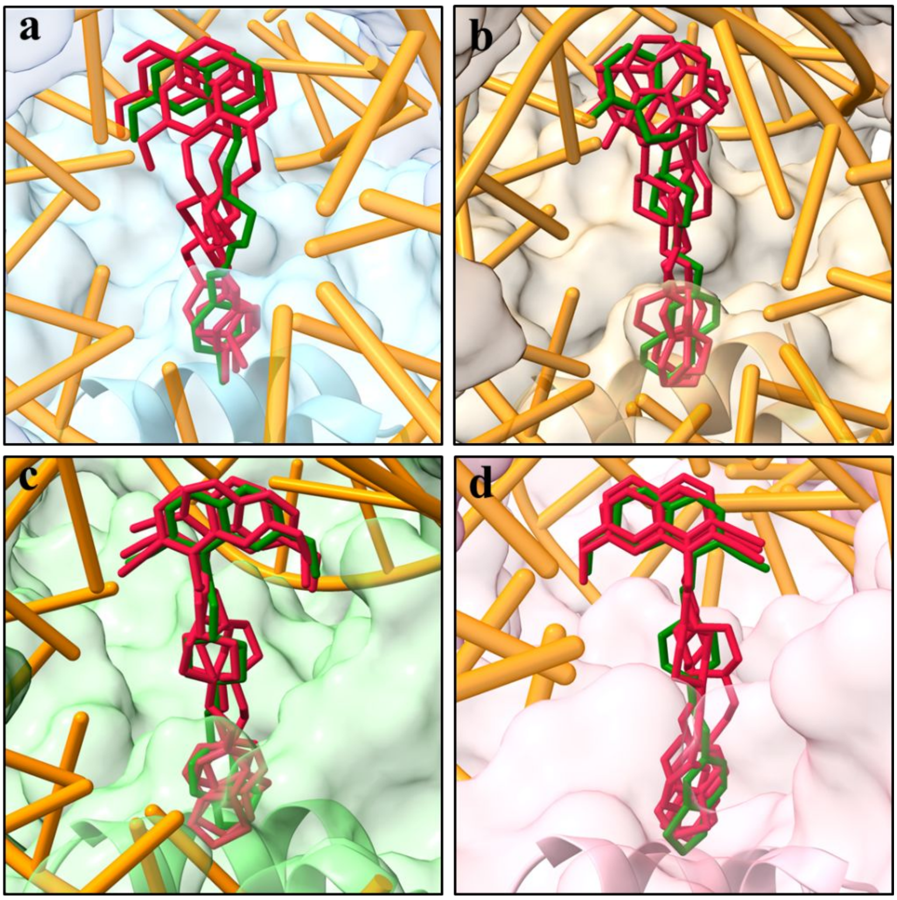

Figure S2: Spatial comparison between natively present ligand conformation and its calculated dock poses in **a)** crystal structure of *S. aureus* DNA gyrase (PDB ID: 6Z1A; AMK-12 ligand),[5] and **b)** cryoEM structure of *E. coli* DNA gyrase (PDB ID: 6RKS, gepotidacin),[6] **c)** homology model *E. coli* topo IV (GSK299423[7]), **d)** homology model *S. aureus* topo IV (GSK299423[7]). The co-crystallized ligand AMK-12 and cryoEM ligand gepotidacin are represented in dark green, while their re-docking derived poses are depicted in solid red. Enzymes are represented as surface and α-helix regions as cartoon.

Table S2: Molecular docking validation data

Protein	RMDS (Å)		
	Docked pose 1	Docked pose 2	Docked pose 3
S. aureus DNA gyrase (PDB ID: 6Z1A; AMK-12)[5]	0.8324	1.7501	1.3039
E. coli DNA gyrase (PDB ID: 6RKS; gepotidacin)[6]	0.9880	1.6198	1.3691
Homology model *E. coli* topo IV (GSK299423[7])	1.2052	1.0780	1.1791
Homology model *S. aureus* topo IV (GSK299423[7])	1.0168	1.4858	1.5951

RMSD values in Angstrom units (Å) obtained by alignment (heavy atoms) of each calculated dock pose and its natively present co-crystallized/cryoEM conformation[5,6] within GyrA, i.e., ParC *S. aureus*/*E. coli*.

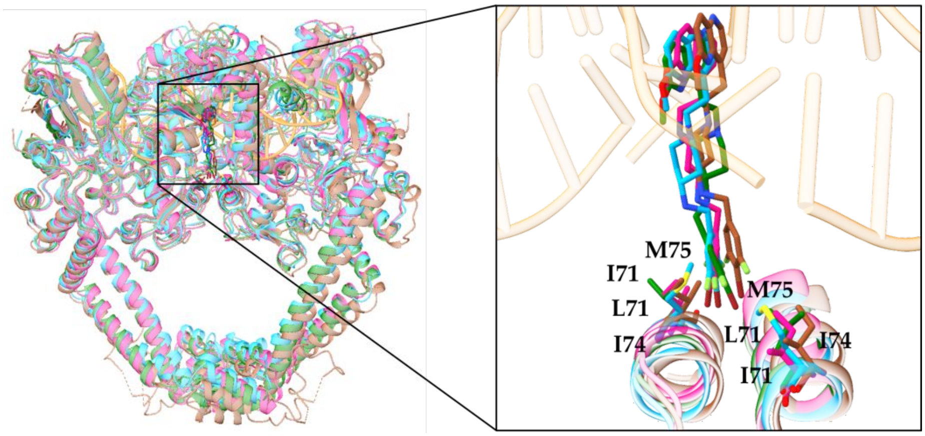

Figure S3: Structural superimposition of all four enzymes and docked poses of compound **8**. *S. aureus* DNA gyrase (blue, PDB ID: 6Z1A)[5], *E. coli* DNA gyrase (brown, PDB ID: 6RKS)[6], *S. aureus* topo IV homology model utilizing obtained by utilizing *Streptococcus pneumoniae* topo IV structure as a template (pink, PDB ID: 3RAF),[2] and *E. coli* topo IV homology model obtained by utilizing *S. pneumoniae* topo IV structure as a template (green, PDB ID: 3KSA)[3]. Docked poses of **8** are inserted artificially and coloured according to the colouring scheme of the corresponding enzymes. Enzymes are shown in cartoon, NBTI **8** poses (sticks representation), GyrA/ParC amino acid residues (sticks representation) are coloured by element, while DNA in orange.

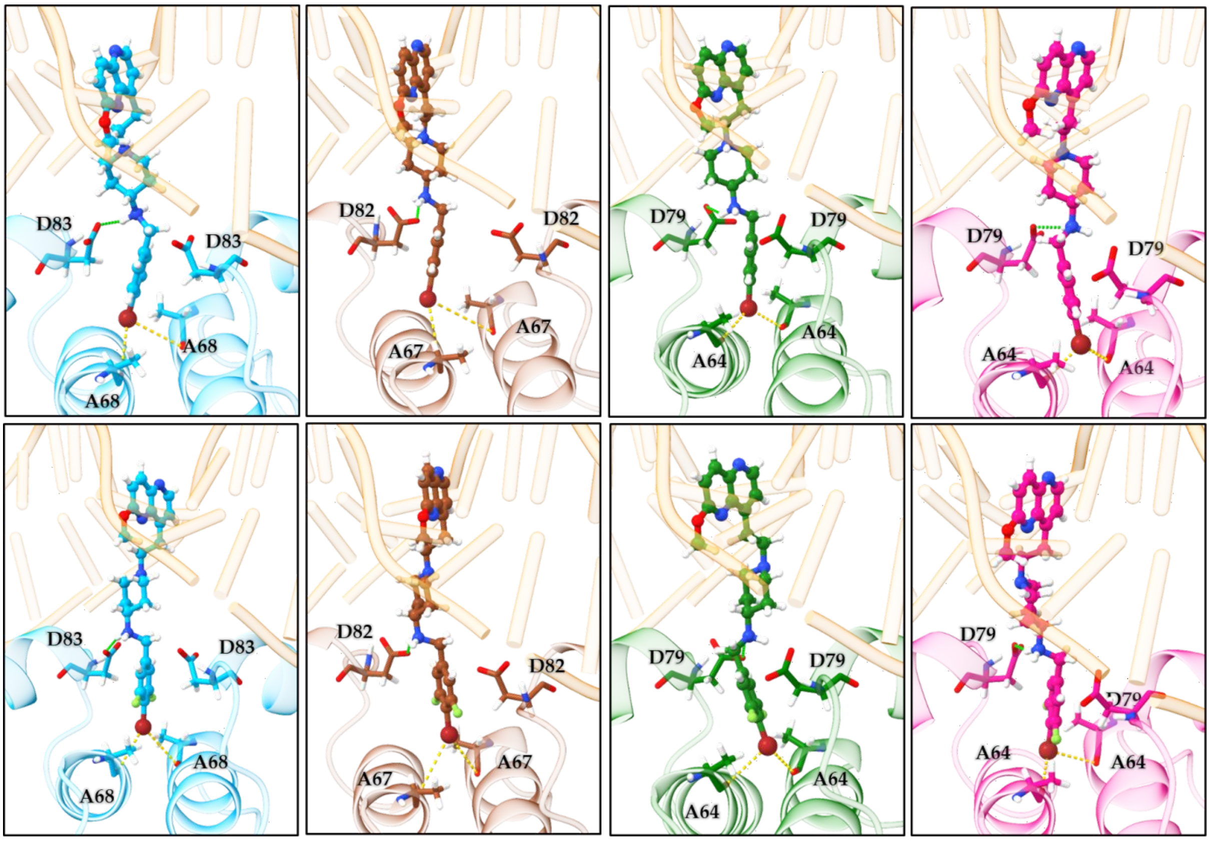

Figure S4: Comparison of predicted binding poses for compounds **2** (figures above) and **8** (figures below) docked in *S. aureus* DNA gyrase (inset blue) and *S. aureus* topoIV (inset pink), i.e., *E. coli* DNA gyrase (inset brown) and *E. coli* topoIV (inset green). GyrA (DNA gyrase), i.e., ParC (topoIV) subunits are in cartoon representation, the ionic interactions between protonated ligand's nitrogen and aspartate residues are shown as light-green dots, while the bifurcated halogen bonds are represented as yellow dots.

General Chemical Methods

Starting materials, reagents and solvents were obtained from commercial sources and used without additional preparation.

Thin layer chromatography (TLC) analysis was used for the progress of the reaction, product isolation and selection of solvents for purification. The analytical TLC was performed on Merck 60 F254 (0.25 mm) silica gel plates, the products were visualized with UV light and spray reagents.

The final products were purified by column chromatography on silica gel 60 (particle size 240-400 mesh). The compounds were eluted with an optimized gradient of dichloromethane - methanol.

Purity and identity were further confirmed by NMR spectroscopy. The resonance frequency for ^{1}H and ^{13}C in NMR was recorded at 400 MHz and 100 MHz with an AVANCE III 400 spectrometer (Bruker Corporation, Billerica, MA, USA) in $CDCl_3$. The chemical shifts (δ) are given in parts per million (ppm) relative to the deuterated solvent as internal standard (δH: $CDCl_3$ 7.26 ppm), the coupling constants (*J*) are in Hertz (Hz). The peak multiplicities are expressed as follows: singlet (s), doublet (d), doublet of doublets (dd), triplet (t), multiplet (m).

High resolution mass spectra were recorded on a LC-MS/MS system (Q Executive Plus; Thermo Scientific, MA, USA).

Unless otherwise indicated, all compounds had a purity ≥ 95 %, determined by HPLC on an 1100 system (Thermo Scientific Dionex UltiMate 3000 (Thermo Fisher Scientific, Inc.). The general method used a Waters Acquity UPLC® HSS C18 SB column (2.1 × 50 mm, 1.8 µm) thermostated at

40 °C, with: injection volume, 5 µL; sample, 0.1–0.2 mg/mL in MeOH; flow rate, 0.4 mL/min; detector λ, 220 and 254 nm; mobile phase A: 0.1% TFA (v/v) in water; mobile phase B: MeCN. Gradient: 0–2 min, 20% B; 2–5 min, 20%–90% B; 5–8 min, 90% B.

Deprotection of NH_2: BOC protect group

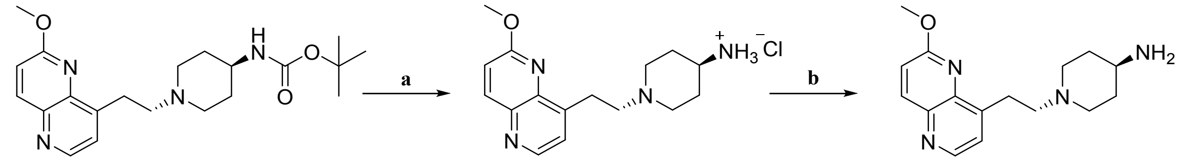

S1 **S2a** **S2b**

Scheme S1: Deprotection of NH_2; a: TFA, MeOH, rt, 2 h; b: 0.1 M NaOH

To the commercially available compound **S1** (DSK-1030, DSK InnoSciences Pvt.Ltd., India) (800 mg, 2.07 mmol, 1 eq) in methanol TFA (3 mL, 39.2 mmol, 19 eq) was added. The reaction mixture was stirred at room temperature for 2 h to obtain **S2a**. To the reaction mixture 0.1 M NaOH (30 mL) was added and the pH was adjusted with 1 M NaOH to approximately 12. The suspension was extracted with 3 × 30 mL DCM. The combined organic phases were dried over Na_2SO_4, filtered and evaporated to afford **S2b** (567 mg, 95.6 %).

General procedure: Reductive amination

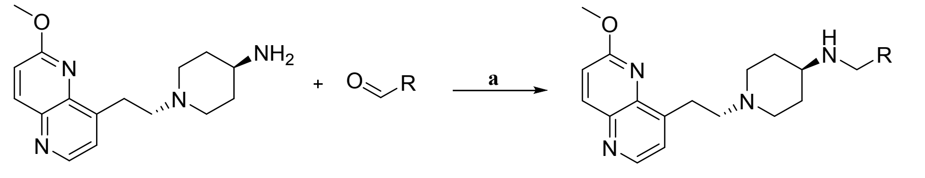

S2b **4 - 13**

4: **5:** **6:** **7:** **8:** **9:** **10:** **11:** **12:** **13:**

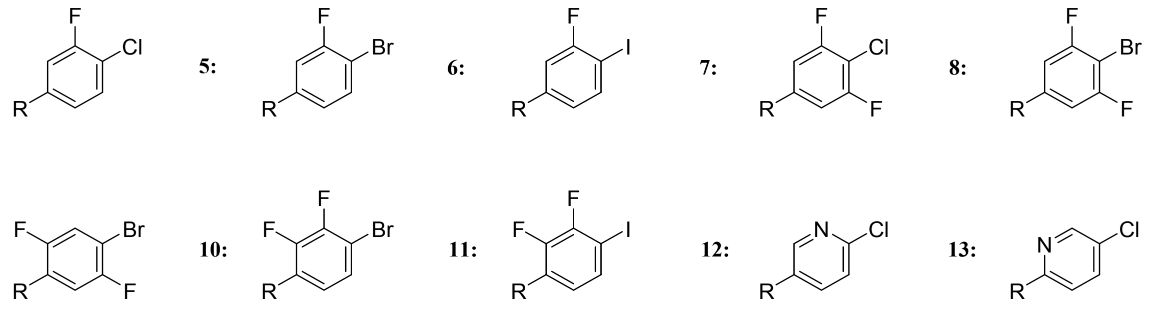

Scheme S2: General procedure – reductive amination; a: 1. AcOH, MeOH, rt, 1 h; b: $NaCNBH_3$, rt, 16 h.

To a solution of **2b** (200 mg, 0.698 mmol, 1 eq) and suitable aldehyde (0.908 mmol, 1.3 eq) in methanol (~ 5 mL) acetic acid (2 drops) was added and the mixture was stirred at room temperature for 1 h. To the reaction mixture $NaCNBH_3$ (175 mg, 2.793 mmol, 4 eq) was added and reaction was stirred at room temperature for 16 h. Then the solvent was evaporated and the crude product was dissolved in ethyl acetate (20 mL) and washed with 3 × 10 mL of 0.1 M NaOH. The organic phase was dried over Na_2SO_4, filtered and evaporated. The crude product was purified by flash chromatography on silica gel to afford the title compounds (**4** - **13**).

Synthetic processes of individual compounds

***N*-(4-chloro-3-fluorobenzyl)-1-(2-(6-methoxy-1, 5-naphthyridin-4-yl)ethyl)piperidin-4-amine (4)**

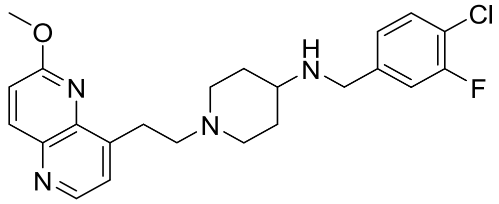

The title compound was obtained according to general procedure using **2b** (200 mg, 0.698 mmol, 1 eq), 4-chloro-3-fluorobenzaldehyde (144 mg, 0.908 mmol, 1.3 eq), acetic acid, methanol (5 mL) and $NaCNBH_3$ (175 mg, 2.793 mmol, 4 eq). The crude product was purified by flash column chromatography (SiO_2, dichlorometane : methanol = 20 : 1 to 9:1) to afford **4** (214 mg, 71.4 %) as an orange resin solid.

^{1}H NMR (400 MHz, $CDCl_3$): δ 8.65 (d, J = 4.5 Hz, 1H, Ar–H), 8.18 (d, J = 9.0 Hz, 1H, Ar–H), 7.41 (d, J = 4.5 Hz, 1H, Ar–H), 7.34 – 7.28 (m, 1H, Ar–H), 7.17 (dd, J = 10.0, 1.8 Hz, 1H, Ar–H), 7.10 (d, J = 9.0 Hz, 1H, Ar–H), 7.05 (dd, J = 8.2, 1.2 Hz, 1Hz, Ar–H), 4.07 (s, 3H, C$\underline{H_3}$–O–Ar), 3.80 (s, 2H, Ar–C$\underline{H_2}$–NH), 3.42 – 3.35 (m, 2H, N–(C$\underline{H_2}$)$_2$–Ar), 3.07 – 3.04 (m, 2H, N(C$\underline{H_2}$CH$_2$)$_2$CH), 2.84 – 2.77 (m, 2H, N–(C$\underline{H_2}$)$_2$–Ar), 2.57 – 2.48 (m, 1H, N(CH$_2$CH$_2$)$_2$C$\underline{H}$), 2.24 – 2.15 (m, 2H, N(C$\underline{H_2}$CH$_2$)$_2$CH), 1.99 – 1.90 (m, 2H, N(CH$_2$C$\underline{H_2}$)$_2$CH), 1.54 – 1.43 (m, 2H, N(CH$_2$C$\underline{H_2}$)$_2$CH) ppm. "Amine H is exchangeable and is not visible on NMR spectra."

^{13}C NMR (100 MHz, $CDCl_3$): δ 161.50 (Ar–C), 158.18 (d, J = 248.5 Hz, Ar–C), 147.77 (Ar–C), 146.59 (Ar–C), 142.13 (d, J = 6.0 Hz, Ar–C), 141.58 (Ar–C), 141.03 (Ar–C), 140.42 (Ar–C), 130.41 (Ar–C), 124.31 (Ar–C), 124.24 (d, J = 3.5 Hz, Ar–C), 119.10 (d, J = 17.7 Hz, Ar–C), 116.36 (Ar–C), 116.20 (Ar–C), 115.99 (Ar–C), 58.42 (N–($\underline{C}$H$_2$)$_2$–Ar), 54.08 (N(CH$_2$CH$_2$)$_2$$\underline{C}$H), 53.77 ($\underline{C}H_3$–O–Ar), 52.29 (N($\underline{C}H_2CH_2$)$_2$CH), 49.79 (Ar–$\underline{C}H_2$–NH), 32.72 (N(CH$_2$$\underline{C}H_2$)$_2$CH), 28.47 (N–($\underline{C}H_2$)$_2$–Ar) ppm.

HRMS: m/z: calcd for $C_{23}H_{27}ClFN_4O$ $[M+H]^+$: 429.1852, found: 429.1841.

HPLC: t_R = 3.587 min (95 % at 254 nm)

***N*-(4-bromo-3-fluorobenzyl)-1-(2-(6-methoxy-1,5-naphthyridin-4-yl)ethyl)piperidin-4-amine (5)**

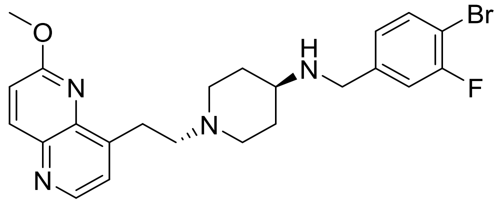

The title compound was obtained according to general procedure using **2b** (167 mg, 0.583 mmol, 1 eq), 4-bromo-3-fluorobenzaldehyde (154 mg, 0.758 mmol, 1.3 eq), acetic acid, methanol (5 mL) and $NaCNBH_3$ (147 mg, 2.333 mmol, 4 eq). The crude product was purified by flash column chromatography (SiO_2, dichlorometane : methanol = 19 : 1 to 4:1) to afford **5** (217 mg, 78.6 %) as a brown solid.

^{1}H NMR (400 MHz, $CDCl_3$): δ 8.63 (d, J = 4.5 Hz, 1H, Ar–H), 8.15 (d, J = 9.0 Hz, 1H, Ar–H), 7.44 (dd, J = 8.1, 7.2 Hz, 1H, Ar–H), 7.38 (d, J = 4.5 Hz, 1H, Ar–H), 7.13 (dd, J = 9.6, 1.9 Hz, 1H, Ar–H), 7.08 (d, J = 9.0 Hz, 1H, Ar–H), 6.98 (dd, J = 8.2, 1.5 Hz, 1H, Ar–H), 4.05 (s, 3H, C$\underline{H_3}$–O–Ar), 3.77 (s, 2H, Ar–C$\underline{H_2}$–NH), 3.38 – 3.33 (m, 2H, N–(C$\underline{H_2}$)$_2$–Ar), 3.05 – 2.99 (m, 2H, N(C$\underline{H_2}$CH$_2$)$_2$CH), 2.80 – 2.74 (m, 2H, N–(C$\underline{H_2}$)$_2$–Ar), 2.54 – 2.46 (m, 1H, N(CH$_2$CH$_2$)$_2$C$\underline{H}$), 2.21 – 2.11 (m, 2H, N(C$\underline{H_2}$CH$_2$)$_2$CH), 1.96 – 1.88 (m, 2H, N(CH$_2$C$\underline{H_2}$)$_2$CH), 1.51 – 1.40 (m, 2H, N(CH$_2$C$\underline{H_2}$)$_2$CH) ppm. "Amine H is exchangeable and is not visible on NMR spectra."

^{13}C NMR (100 MHz, $CDCl_3$): δ 161.49 (Ar–C), 159.17 (d, J = 247.2 Hz, Ar–C), 147.76 (Ar–C), 146.63 (Ar–C), 143.05 (d, J = 6.3 Hz, Ar–C), 141.56 (Ar–C), 141.03 (Ar–C), 140.41 (Ar–C), 133.30 (Ar–C), 124.73 (d, J = 3.5 Hz, Ar–C), 124.31 (Ar–C), 116.36 (Ar–C), 116.01 (d, J = 22.2 Hz, Ar–C), 106.94 (d, J = 21.1 Hz, Ar–C), 58.43 (N–($\underline{C}H_2)_2$–Ar), 54.09 ($N(CH_2CH_2)_2\underline{C}H$), 53.77 ($\underline{C}H_3$–O–Ar), 52.30 ($N(\underline{C}H_2CH_2)_{\underline{2}}CH$), 49.80 (Ar–$\underline{C}H_2$–NH), 32.75 ($N(CH_2\underline{C}H_2)_{\underline{2}}CH$), 28.48 (N–($\underline{C}H_2)_2$–Ar) ppm.

HRMS: m/z: calcd for $C_{23}H_{27}BrFN_4O$ $[M+H]^+$: 473.1347, found: 473.1338.

HPLC: t_R = 3.610 min (96 % at 254 nm)

***N*-(3-fluoro-4-iodobenzyl)-1-(2-(6-methoxy-1,5-naphthyridin-4-yl)ethyl)piperidin-4-amine (6)**

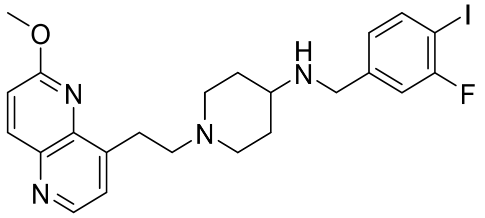

The title compound was obtained according to general procedure using **2b** (182 mg, 0.636 mmol, 1 eq), 3-fluoro-4-iodobenzaldehyde (207 mg, 0.826 mmol, 1.3 eq), acetic acid, methanol (5 mL) and $NaCNBH_3$ (160 mg, 2.542mmol, 4 eq). The crude product was purified by flash column chromatography (SiO_2, dichlorometane : methanol = 19 : 1 to 4:1) to afford **6** (133 mg, 40.2 %) as a yellow solid.

^{1}H NMR (400 MHz, $CDCl_3$): δ 8.65 (d, J = 4.4 Hz, 1H, Ar–H), 8.18 (d, J = 9.0 Hz, 1H, Ar–H), 7.66 (t, J = 7.3 Hz, 1H, Ar–H), 7.41 (d, J = 4.4 Hz, 1H, Ar–H), 7.10 (d, J = 9.0 Hz, 2H, Ar–H), 6.89 (d, J = 8.0 Hz, 1H, Ar–H), 4.07 (s, 3H, $C\underline{H_3}$–O–Ar), 3.80 (s, 2H, Ar–$C\underline{H_2}$–NH), 3.44 – 3.33 (m, 2H, N–($C\underline{H_2})_2$–Ar), 3.14 – 2.97 (m, 2H, $N(C\underline{H_2}CH_2)_2CH$), 2.85 – 2.74 (m, 2H, N–($C\underline{H_2})_2$–Ar), 2.58 – 2.46 (m, 1H, $N(CH_2CH_2)_2C\underline{H}$), 2.29 – 2.12 (m, 2H, $N(C\underline{H_2}CH_2)_2CH$), 2.01 – 1.89 (m, 2H, $N(CH_2C\underline{H_2})_2CH$), 1.55 – 1.41 (m, 2H, $N(CH_2C\underline{H_2})_2CH$) ppm. "Amine H is exchangeable and is not visible on NMR spectra."

^{13}C NMR (100 MHz, $CDCl_3$): δ 161.79 (d, J = 6.3 Hz, Ar–C), 161.41 (Ar–C), 147.69 (Ar–C), 146.52 (Ar–C), 144.20 (d, J = 6.3 Hz, Ar–C), 141.49 (Ar–C), 140.95 (Ar–C), 140.34 (Ar–C), 139.05 (Ar–C), 125.26 (d, J = 3.2 Hz, Ar–C), 124.23 (Ar–C), 116.27 (Ar–C), 115.17 (d, J = 23.7 Hz, Ar–C), 78.68 (d, J = 25.6 Hz, Ar–C), 58.34 (N–($\underline{C}H_2)_2$–Ar), 53.96 ($N(CH_2CH_2)2\underline{C}H$), 53.69 ($\underline{C}H_3$–O–Ar), 52.20 ($N(\underline{C}H_2CH_2)_{\underline{2}}CH$), 49.72 (Ar–$\underline{C}H_2$–NH), 32.65 ($N(CH_2\underline{C}H_2)_{\underline{2}}CH$), 28.39 (N–($\underline{C}H_2)_2$–Ar) ppm.

HRMS: m/z: calcd for $C_{23}H_{27}IFN_4O$ $[M+H]^+$: 521.1208, found: 521.1199.

HPLC: t_R = 3.663 min (97 % at 254 nm)

***N*-(4-chloro-3,5-difluorobenzyl)-1-(2-(6-methoxy-1,5-naphthyridin-4-yl)ethyl)piperidin-4-amine (7)**

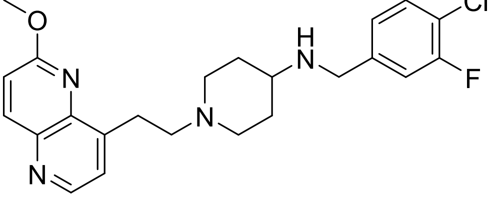

The title compound was obtained according to general procedure using **2b** (200 mg, 0.698 mmol, 1 eq), 4-chloro-3,5-difluorobenzaldehyde (160 mg, 0.908 mmol, 1.3 eq), acetic acid, methanol (5 mL) and $NaBH(OAc)_3$ (222 mg, 1.047, 1.5 eq). The crude product was purified by flash column chromatography (SiO_2, dichlorometane : methanol = 19 : 1 to 4:1) to afford **7** (131 mg, 42.0 %) as an orange resin solid.

^{1}H NMR (400 MHz, $CDCl_3$): δ 8.63 (d, J = 4.5 Hz, 1H, Ar–H), 8.15 (d, J = 9.0 Hz, 1H, Ar–H), 7.38 (d, J = 4.5 Hz, 1H, Ar–H), 7.08 (d, J = 9.0 Hz, 1H, Ar–H), 6.98 (d, J = 7.6 Hz, 2H, Ar–H), 4.05 (s, 3H, C$\underline{H_3}$–O–Ar), 3.77 (s, 2H, Ar–C$\underline{H_2}$–NH), 3.38 – 3.32 (m, 2H, N–(C$\underline{H_2}$)$_2$–Ar), 3.06 – 2.98 (m, 2H, N(C$\underline{H_2}$CH$_2$)$_2$CH), 2.79 – 2.73 (m, 2H, N–(C$\underline{H_2}$)$_2$–Ar), 2.52 – 2.44 (m, 1H, N(CH$_2$CH$_2$)$_2$C$\underline{H}$), 2.20 – 2.11 (m, 2H, N(C$\underline{H_2}$CH$_2$)$_2$CH), 1.94 – 1.86 (m, 2H, N(CH$_2$C$\underline{H_2}$)$_2$CH), 1.50 – 1.39 (m, 2H, N(CH$_2$C$\underline{H_2}$)$_2$CH) ppm. "Amine H is exchangeable and is not visible on NMR spectra."

^{13}C NMR (100 MHz, $CDCl_3$): δ 161.50 (Ar–C), 158.91 (dd, J = 250.1, 3.6 Hz, Ar–C), 147.78 (Ar–C), 146.67 (Ar–C), 142.53 (t, J = 7.7 Hz, Ar–C), 141.58 (Ar–C), 141.05 (Ar–C), 140.43 (Ar–C), 124.30 (Ar–C), 116.35 (Ar–C), 111.30 (Ar–C), 111.10 (Ar–C), 107.71 (t, J = 21.2 Hz, Ar–C), 58.46 (N–($\underline{C}$H$_2$)$_2$–Ar), 54.21 (N(CH$_2$CH$_2$)$_2\underline{C}$H), 53.76 ($\underline{C}$H$_3$–O–Ar), 52.34 (N($\underline{C}$H$_2$CH$_2$)$_2$CH), 49.69 (Ar–$\underline{C}$H$_2$–NH), 32.87 (N(CH$_2\underline{C}$H$_2$)$_2$CH), 28.54 (N–($\underline{C}$H$_2$)$_2$–Ar) ppm.

HRMS: m/z: calcd for $C_{23}H_{26}ClF_2N_4O$ $[M+H]^+$: 447.1758, found: 447.1747.

HPLC: t_R = 3.697 min (99 % at 254 nm)

***N*-(4-bromo-3, 5-difluorobenzyl)-1-(2-(6-methoxy-1,5-naphthyridin-4-yl)ethyl)piperidin-4-amine (8)**

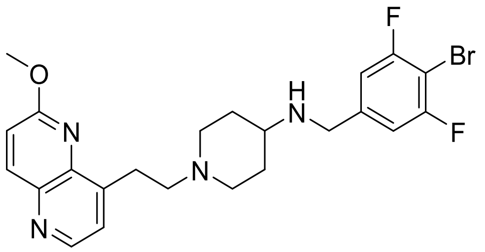

The title compound was obtained according to general procedure using **2b** (188 mg, 0.656 mmol, 1 eq), 4-bromo-3,5-difluorobenzaldehyde (189 mg, 0.853 mmol, 1.3 eq), acetic acid, methanol (5 mL) and $NaCNBH_3$ (165 mg, 2.629 mmol, 4 eq). The crude product was purified by flash column chromatography (SiO_2, dichlorometane : methanol = 19 : 1 to 4:1) to afford **8** (239 mg, 74.1 %) as a light brown solid.

^{1}H NMR (400 MHz, $CDCl_3$): δ 8.63 (d, J = 4.5 Hz, 1H, Ar–H), 8.16 (d, J = 9.0 Hz, 1H, Ar–H), 7.39 (d, J = 4.5 Hz, 1H, Ar–H), 7.08 (d, J = 9.0 Hz, 1H, Ar–H), 6.96 (d, J = 7.3 Hz, 2H, Ar–H), 4.05 (s, 3H, C$\underline{H_3}$–O–Ar), 3.78 (s, 2H, Ar–C$\underline{H_2}$–NH), 3.39 – 3.32 (m, 2H, N–(C$\underline{H_2}$)$_2$–Ar), 3.05 – 3.00 (m, 2H, N(C$\underline{H_2}$CH$_2$)$_2$CH), 2.81 – 2.75 (m, 2H, N–(C$\underline{H_2}$)$_2$–Ar), 2.53 – 2.44 (m, 1H, N(CH$_2$CH$_2$)$_2$C$\underline{H}$), 2.21 – 2.11 (m, 2H, N(C$\underline{H_2}$CH$_2$)$_2$CH), 1.95 – 1.87 (m, 2H, N(CH$_2$C$\underline{H_2}$)$_2$CH), 1.51 – 1.39 (m, 2H, N(CH$_2$C$\underline{H_2}$)$_2$CH) ppm. "Amine H is exchangeable and is not visible on NMR spectra."^{13}C NMR (100 MHz, $CDCl_3$): δ 161.51 (Ar–C), 161.20 (d, J = 4.4 Hz, Ar–C), 158.73 (d, J = 4.5 Hz, Ar–C), 147.78 (Ar–C), 146.63 (Ar–C), 143.81 (t, J = 8.0 Hz, Ar–C), 141.59 (Ar–C), 141.05 (Ar–C), 140.44 (Ar–C), 124.31 (Ar–C), 116.37 (Ar–C), 111.30 (d, J = 2.4 Hz, Ar–C), 111.07 (d, J = 2.6 Hz, Ar–C), 95.63 (t, J = 24.6 Hz, Ar–C), 58.45 (N–($\underline{C}$H$_2$)$_2$–Ar), 54.15 (N(CH2CH$_2$)$_2\underline{C}$H), 53.78 ($\underline{C}$H$_3$–O–Ar), 52.32 (N($\underline{C}$H$_2$CH$_2$)$_2$CH), 49.71 (Ar–$\underline{C}$H$_2$–NH), 32.82 (N(CH$_2\underline{C}$H$_2$)$_2$CH), 28.52 (N–($\underline{C}$H$_2$)$_2$–Ar) ppm.

HRMS: m/z: calcd for $C_{23}H_{26}BrF_2N_4O$ $[M+H]^+$: 491.1253, found: 491.1241.

HPLC: t_R = 3.693 min (95 % at 254 nm)

N-(4-bromo-2, 5-difluorobenzyl)-1-(2-(6-methoxy-1,5-naphthyridin-4-yl)ethyl)piperidin-4-amine (9)

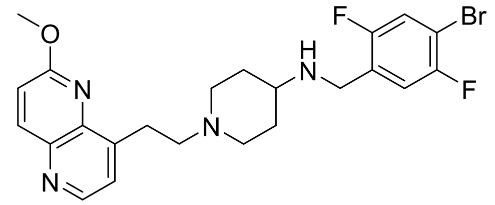

The title compound was obtained according to general procedure using **2b** (188 mg, 0.656 mmol, 1 eq), 4-bromo-2,5-difluorobenzaldehyde (189 mg, 0.853 mmol, 1.3 eq), acetic acid, methanol (5 mL) and $NaCNBH_3$ (165 mg, 2.629 mmol, 4 eq). The crude product was purified by flash column chromatography (SiO_2, dichlorometane : methanol = 19 : 1 to 4:1) to afford **9** (212 mg, 65.6 %) as a yellow solid.

^{1}H NMR (400 MHz, $CDCl_3$): δ 8.63 (d, J = 4.5 Hz, 1H, Ar–H), 8.16 (d, J = 9.0 Hz, 1H, Ar–H), 7.39 (d, J = 4.5 Hz, 1H, Ar–H), 7.24 – 7.18 (m, 2H, Ar–H), 7.09 (d, J = 9.0 Hz, 1H, Ar–H), 4.05 (s, 3H, $C\underline{H_3}$–O–Ar), 3.80 (s, 2H, Ar–$C\underline{H_2}$–NH), 3.40 – 3.32 (m, 2H, N–$(C\underline{H_2})_2$–Ar), 3.08 – 2.98 (m, 2H, $N(C\underline{H_2}CH_2)_2CH$), 2.81 – 2.75 (m, 2H, N–$(C\underline{H_2})_2$–Ar), 2.54 – 2.45 (m, 1H, $N(CH_2CH_2)_2C\underline{H}$), 2.22 – 2.14 (m, 2H, $N(C\underline{H_2}CH_2)_2CH$), 1.98 – 1.89 (m, 2H, $N(CH_2C\underline{H_2})_2CH$), 1.53 – 1.40 (m, 2H, $N(CH_2C\underline{H_2})_2CH$) ppm. "Amine H is exchangeable and is not visible on NMR spectra."^{13}C NMR (100 MHz, $CDCl_3$): δ 161.41 (Ar–C), 157.17 (dd, J = 77.1, 2.8 Hz, Ar–C), 154.74 (dd, J = 75.3, 2.7 Hz, Ar–C), 147.67 (Ar–C), 146.57 (Ar–C), 141.48 (Ar–C), 140.95 (Ar–C), 140.32 (Ar–C), 129.26 (dd, J = 17.2, 6.1 Hz, Ar–C), 124.22 (Ar–C), 119.90 (d, J = 27.3 Hz, Ar–C), 116.88 (dd, J = 24.9, 5.7 Hz, Ar–C), 116.28 (Ar–C), 106.83 (dd, J = 24.0, 10.3 Hz, Ar–C), 58.35 (N–$(\underline{C}H_2)_2$–Ar), 54.02 ($N(CH_2CH_2)_2\underline{C}H$), 53.68 ($\underline{C}H_3$–O–Ar), 52.17 ($N(\underline{C}H_2CH_2)_{\underline{2}}CH$), 43.35 (Ar–$\underline{C}H_2$–NH), 32.64 ($N(CH_2\underline{C}H_2)_{\underline{2}}CH$), 28.41 (N–$(\underline{C}H_2)_2$–Ar) ppm.

ESI-MS: m/z: calcd for $C_{23}H_{26}BrF_2N_4O$ $[M+H]^+$: 491.1253, found: 491.1242

HPLC: t_R = 3.570 min (96 % at 254 nm)

***N*-(4-bromo-2,3-difluorobenzyl)-1-(2-(6-methoxy-1,5-naphthyridin-4-yl)ethyl)piperidin-4-amine (10)**

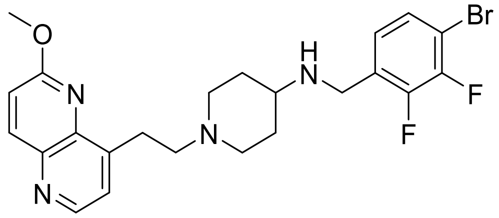

The title compound was obtained according to general procedure using **2b** (170 mg, 0.594 mmol, 1 eq), 4-bromo-2,3-difluorobenzaldehyde (171 mg, 0.772 mmol, 1.3 eq), acetic acid, methanol (5 mL) and $NaCNBH_3$ (150 mg, 2.374 mmol, 4 eq). The crude product was purified by flash column chromatography (SiO_2, dichlorometane : methanol = 19 : 1 to 4:1) to afford **10** (239 mg, 81.2 %) as a light brown solid.

^{1}H NMR (400 MHz, $CDCl_3$): δ 8.65 (d, J = 4.5 Hz, 1H, Ar–H), 8.20 – 8.16 (m, 1H, Ar–H), 7.41 (d, J = 4.5 Hz, 1H, Ar–H), 7.29 – 7.24 (m, 1H, Ar–H), 7.10 (d, J = 9.0 Hz, 1H, Ar–H), 7.08 – 7.05 (m, 1H, Ar–H), 4.07 (s, 3H, $C\underline{H_3}$–O–Ar), 3.87 (s, 2H, Ar–$C\underline{H_2}$–NH), 3.41 – 3.35 (m, 2H, N–$(C\underline{H_2})_2$–Ar), 3.08 – 3.01 (m, 2H, $N(C\underline{H_2}CH_2)_2CH$), 2.83 – 2.77 (m, 2H, N–$(C\underline{H_2})_2$–Ar), 2.56 – 2.47 (m, 1H, $N(CH_2CH_2)_2C\underline{H}$), 2.24 – 2.15 (m, 2H, $N(C\underline{H_2}CH_2)_2CH$), 1.98 – 1.90 (m, 2H, $N(CH_2C\underline{H_2})_2CH$), 1.55 –

1.43 (m, 2H, N(CH$_2$C$\underline{H_2}$)$_2$CH) ppm. "Amine H is exchangeable and is not visible on NMR spectra."^{13}C NMR (100 MHz, CDCl$_3$): δ 161.50 (Ar–C), 150.62 (d, J = 13.3 Hz, Ar–C), 149.25 (d, J = 14.7 Hz, Ar–C), 148.13 (d, J = 13.6 Hz, Ar–C), 147.77 (Ar–C), 146.64 (Ar–C), 141.58 (Ar–C), 141.04 (Ar–C), 140.43 (Ar–C), 129.75 (d, J = 12.3 Hz, Ar–C), 127.65 (d, J = 4.2 Hz, Ar–C), 125.08 (t, J = 4.2 Hz, Ar–C), 124.31 (Ar–C), 116.35 (Ar–C), 108.45 (d, J = 17.7 Hz, Ar–C), 58.43 (N–($\underline{C}$H$_2$)$_2$–Ar), 54.03 (N(CH$_2$CH$_2$)$_2$$\underline{C}$H), 53.77 ($\underline{C}H_3$–O–Ar), 52.21 (N($\underline{C}H_2CH_2$)$_2$CH), 43.56 (Ar–$\underline{C}H_2$–NH), 32.68 (N(CH$_2$$\underline{C}H_2$)$_2$CH), 28.49 (N–($\underline{C}H_2$)$_2$–Ar) ppm.

HRMS: m/z: calcd for $C_{23}H_{26}BrF_2N_4O$ [M+H]$^+$: 491.1253, found: 491.1242.

HPLC: t_R = 3.630 min (96 % at 254 nm)

***N*-(2,3-difluoro-4-iodobenzyl)-1-(2-(6-methoxy-1,5-naphthyridin-4-yl)ethyl)piperidin-4-amine (11)**

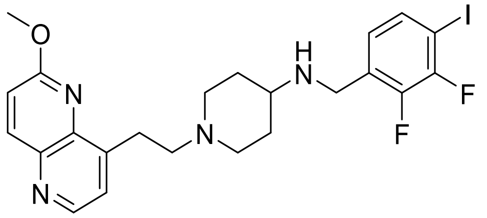

The title compound was obtained according to general procedure using **2b** (202 mg, 0.705 mmol, 1 eq), 2,3-difluoro-4-iodobenzaldehyde (246 mg, 0.917 mmol, 1.3 eq), acetic acid methanol (5 mL) and NaCNBH$_3$ (177 mg, 2.821 mmol, 4 eq). The crude product was purified by flash column chromatography (SiO$_2$, dichlorometane : methanol = 19 : 1 to 9:1) to afford **11** (67 mg, 23.8 %) as a yellow solid.

^{1}H NMR (400 MHz, CDCl$_3$): δ 8.65 (d, J = 4.5 Hz, 1H, Ar–H), 8.17 (d, J = 9.0 Hz, 1H, Ar–H), 7.44 (ddd, J = 10.3, 6.1, 3.4 Hz, 1H, Ar–H), 7.40 (d, J = 4.5 Hz, 1H, Ar–H), 7.10 (d, J = 9.0 Hz, 1H, Ar–H), 7.00 – 6.92 (m, 1H, Ar–H), 4.07 (s, 3H, C$\underline{H_3}$–O–Ar), 3.87 (s, 2H, Ar–C$\underline{H_2}$–NH,), 3.39 – 3.33 (m, 2H, N–(C$\underline{H_2}$)$_2$–Ar), 3.06 – 2.99 (m, 2H, N(C$\underline{H_2}$CH$_2$)$_2$CH), 2.80 – 2.74 (m, 2H, N–(C$\underline{H_2}$)$_2$–Ar), 2.55 – 2.44 (m, 1H, N(CH$_2$CH$_2$)$_2$C$\underline{H}$), 2.20 – 2.11 (m, 2H, N(C$\underline{H_2}$CH$_2$)$_2$CH), 1.95 – 1.87 (m, 2H, N(CH$_2$C$\underline{H_2}$)$_2$CH), 1.52 – 1.40 (m, 2H, N(CH$_2$C$\underline{H_2}$)$_2$CH) ppm. "Amine H is exchangeable and is not visible on NMR spectra."

^{13}C NMR (100 MHz, CDCl$_3$): δ 161.40 (Ar–C), 150.63 (dd, J = 190.9, 14.6 Hz, Ar–C), 148.16 (dd, J = 196.0, 14.6 Hz, Ar–C), 147.69 (Ar–C), 146.73 (Ar–C), 141.50 (Ar–C), 140.98 (Ar–C), 140.35 (Ar–C), 133.19 (d, J = 4.3 Hz, Ar–C), 130.84 (d, J = 12.3 Hz, Ar–C), 126.04 (t, J = 3.8 Hz, Ar–C), 124.20 (Ar–C), 116.25 (Ar–C), 79.95 (d, J = 22.4 Hz, Ar–C), 58.41 (N–($\underline{C}$H$_2$)$_2$–Ar), 54.08 (N(CH$_2$CH$_2$)$_2$$\underline{C}$H), 53.68 ($\underline{C}H_3$–O–Ar), 52.23 (N($\underline{C}H_2CH_2$)$_2$CH), 43.46 (Ar–$\underline{C}H_2$–NH), 32.75 (N(CH$_2$$\underline{C}H_2$)$_2$CH), 28.49 (N–($\underline{C}H_2$)$_2$–Ar) ppm.

HRMS: m/z: calcd for $C_{23}H_{26}IF_2N_4O$ [M+H]$^+$: 539.1114 found: 539.1104.

HPLC: t_R = 3.727 min (98 % at 254 nm)

***N*-((6-chloropyridin-3-yl)methyl)-1-(2-(6-methoxy-1,5-naphthyridin-4-yl)ethyl)piperidin-4-amine (12)**

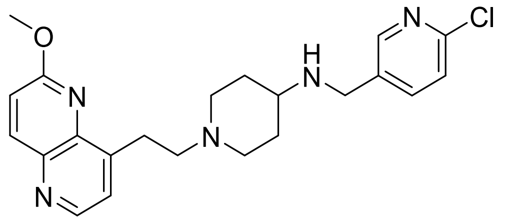

The title compound was obtained according to general procedure using **2b** (208 mg, 0.726 mmol, 1 eq), 6-chloronicotinaldehyde (134 mg, 0.944 mmol, 1.3 eq), acetic acid, methanol (5 mL) and $NaCNBH_3$ (183 mg, 2.905 mmol, 4 eq). The crude product was purified by flash column chromatography (SiO_2, dichlorometane : methanol = 19 : 1 to 3:1) to afford **12** (247 mg, 82.6 %) as a brown resin solid.

^{1}H NMR (400 MHz, $CDCl_3$): δ 8.65 (d, J = 4.5 Hz, 1H, Ar–H), 8.33 (d, J = 2.0 Hz, 1H, Ar–H), 8.20 – 8.15 (m, 1H, Ar–H), 7.69 (dd, J = 8.2, 2.5 Hz, 1H, Ar–H), 7.41 (d, J = 4.5 Hz, 1H, Ar–H), 7.29 (d, J = 8.2 Hz, 1H, Ar–H), 7.12 – 7.09 (m, 1H, Ar–H), 4.07 (s, 3H, $C\underline{H}_3$–O–Ar), 3.83 (s, 2H, Ar–$C\underline{H}_2$–NH), 3.42 – 3.34 (m, 2H, N–$(C\underline{H}_2)_2$–Ar), 3.10 – 3.03 (m, 2H, $N(C\underline{H}_2CH_2)_2CH$), 2.84 – 2.76 (m, 2H, N–$(C\underline{H}_2)_2$–Ar), 2.57 – 2.48 (m, 1H, $N(CH_2CH_2)_2C\underline{H}$), 2.23 – 2.14 (m, 2H, $N(C\underline{H}_2CH_2)_2CH$), 1.98 – 1.91 (m, 2H, $N(CH_2C\underline{H}_2)_2CH$), 1.53 – 1.43 (m, 2H, $N(CH_2C\underline{H}_2)_2CH$) ppm. "Amine H is exchangeable and is not visible on NMR spectra."

^{13}C NMR (100 MHz, $CDCl_3$): δ 161.48 (Ar–C), 150.04 (Ar–C), 149.29 (Ar–C), 147.72 (Ar–C), 146.60 (Ar–C), 141.52 (Ar–C), 141.00 (Ar–C), 140.36 (Ar–C), 138.78 (Ar–C), 135.27 (Ar–C), 124.28 (Ar–C), 124.09 (Ar–C), 116.35 (Ar–C), 58.38 (N–$(\underline{C}H_2)_2$–Ar), 54.20 ($N(CH_2CH_2)_2\underline{C}H$), 53.75 ($\underline{C}H_3$–O–Ar), 52.27 ($N(\underline{C}H_2CH_2)_{\underline{2}}CH$), 47.35 (Ar–$\underline{C}H_2$–NH), 32.72 ($N(CH_2\underline{C}H_2)_{\underline{2}}CH$), 28.45 (N–$(\underline{C}H_2)_2$–Ar) ppm.

HRMS: m/z: calcd for $C_{22}H_{27}ClN_5O$ $[M+H]^+$: 412.1899, found: 412.1891.

HPLC: t_R = 3.177 min (97 % at 254 nm)

***N*-((5-chloropyridin-2-yl)methyl)-1-(2-(6-methoxy-1,5-naphthyridin-4-yl)ethyl)piperidin-4-amin (13)**

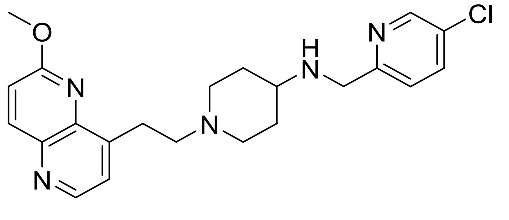

The title compound was obtained according to general procedure using **2b** (166 mg, 0.580 mmol, 1 eq), 5-chloropyridine-2-carboxyaldehyde (107 mg, 0.754 mmol, 1.3 eq), acetic acid, methanol (5 mL) and $NaCNBH_3$ (146 mg, 2.319 mmol, 4 eq). The crude product was purified by flash column chromatography (SiO_2, dichlorometane : methanol = 19 : 1 to 3:1) to afford **13** (96.9 mg, 40.6 %) as a dark brown resin solid.

^{1}H NMR (400 MHz, $CDCl_3$): δ 8.64 (d, J = 4.5 Hz, 1H, Ar–H), 8.49 (d, J = 2.5 Hz, 1H, Ar–H), 8.16 (d, J = 9.0 Hz, 1H, Ar–H), 7.60 (dd, J = 8.3, 2.4 Hz, 1H, Ar–H), 7.39 (d, J = 4.5 Hz, 1H, Ar–H), 7.30 (d, J = 8.2 Hz, 1H, Ar–H), 7.09 (d, J = 9.0 Hz, 1H, Ar–H), 4.06 (s, 3H, $C\underline{H}_3$–O–Ar), 3.92 (s, 2H, Ar–$C\underline{H}_2$–NH), 3.38 – 3.33 (m, 2H, N–$(C\underline{H}_2)_2$–Ar), 3.06 – 3.00 (m, 2H, $N(C\underline{H}_2CH_2)_2CH$), 2.79 – 2.73 (m, 2H, N–$(C\underline{H}_2)_2$–Ar), 2.57 – 2.49 (m, 1H, $N(CH_2CH_2)_2C\underline{H}$), 2.18 – 2.11 (m, 2H, $N(C\underline{H}_2CH_2)_2CH$), 1.96 – 1.90 (m, 2H, $N(CH_2C\underline{H}_2)_2CH$), 1.54 – 1.43 (m, 2H, $N(CH_2C\underline{H}_2)_2CH$) ppm. "Amine H is exchangeable and is not visible on NMR spectra."^{13}C NMR (100 MHz, $CDCl_3$): δ 161.52 (Ar–C),

158.65 (Ar–C), 148.17 (Ar–C), 147.81 (Ar–C), 146.91 (Ar–C), 141.61 (Ar–C), 141.11 (Ar–C), 140.46 (Ar–C), 136.28 (Ar–C), 130.25 (Ar–C), 124.33 (Ar–C), 123.16 (Ar–C), 116.37 (Ar–C), 58.57 (N–($\underline{C}H_2$)$_2$–Ar), 53.80 (N(CH_2CH_2)$_2\underline{C}$H), 52.47 ($\underline{C}H_3$–O–Ar), 51.81 (N($\underline{C}H_2CH_2$)$_{\underline{2}}$CH), 45.60 (Ar–$\underline{C}H_2$–NH), 32.97 (N($CH_2\underline{C}H_2$)$_{\underline{2}}$CH), 28.61 (N–($\underline{C}H_2$)$_2$–Ar) ppm.

HRMS: m/z: calcd for $C_{22}H_{27}ClN_5O$ $[M+H]^+$: 412.1899, found: 412.1890.

HPLC: t_R = 3.323 min (93 % at 254 nm)

NMR spectra

***N*-(4-chloro-3-fluorobenzyl)-1-(2-(6-methoxy-1, 5-naphthyridin-4-yl)ethyl)piperidin-4-amine (4)**

^{1}H NMR (400 MHz, $CDCl_3$)

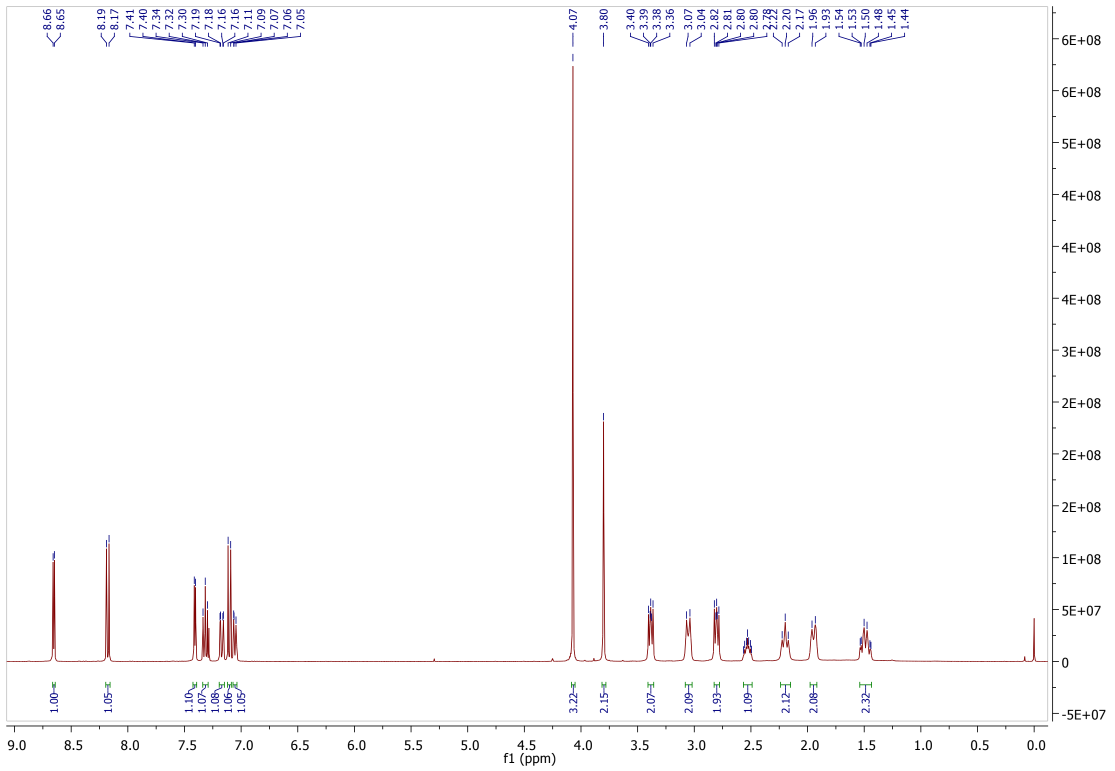

^{13}C NMR (100 MHz, $CDCl_3$)

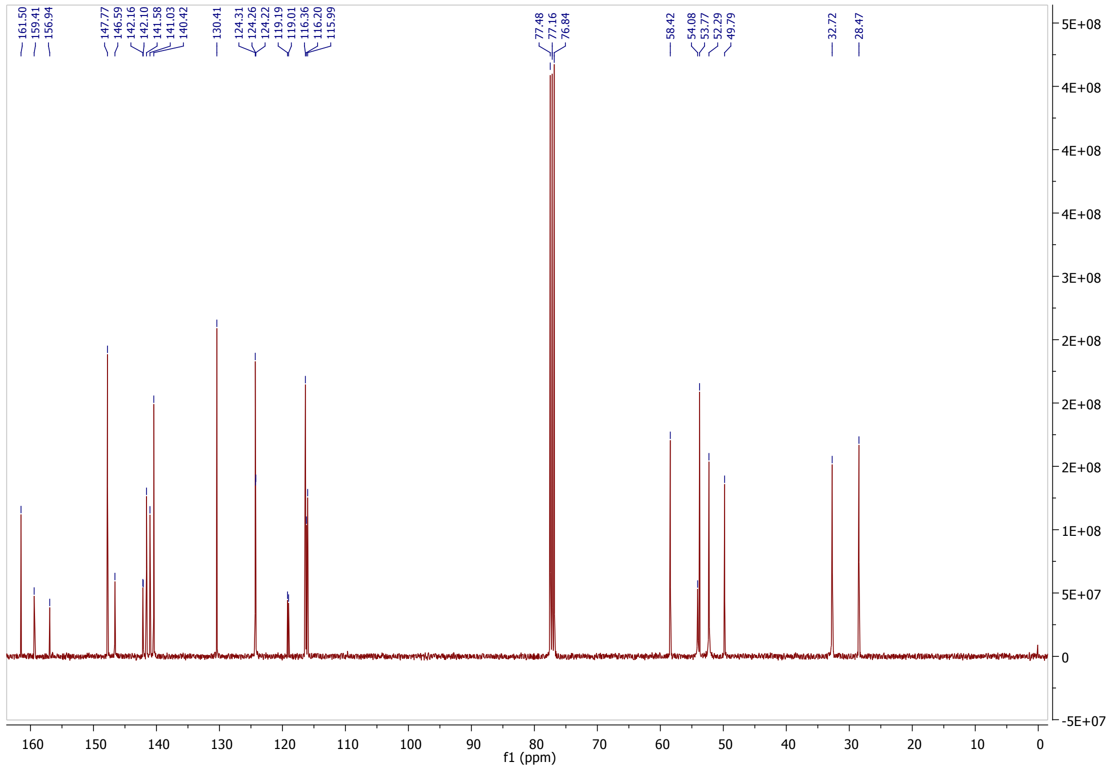

_N_-(4-bromo-3-fluorobenzyl)-1-(2-(6-methoxy-1,5-naphthyridin-4-yl)ethyl)piperidin-4-amine (5)

^{1}H NMR (400 MHz, $CDCl_3$)

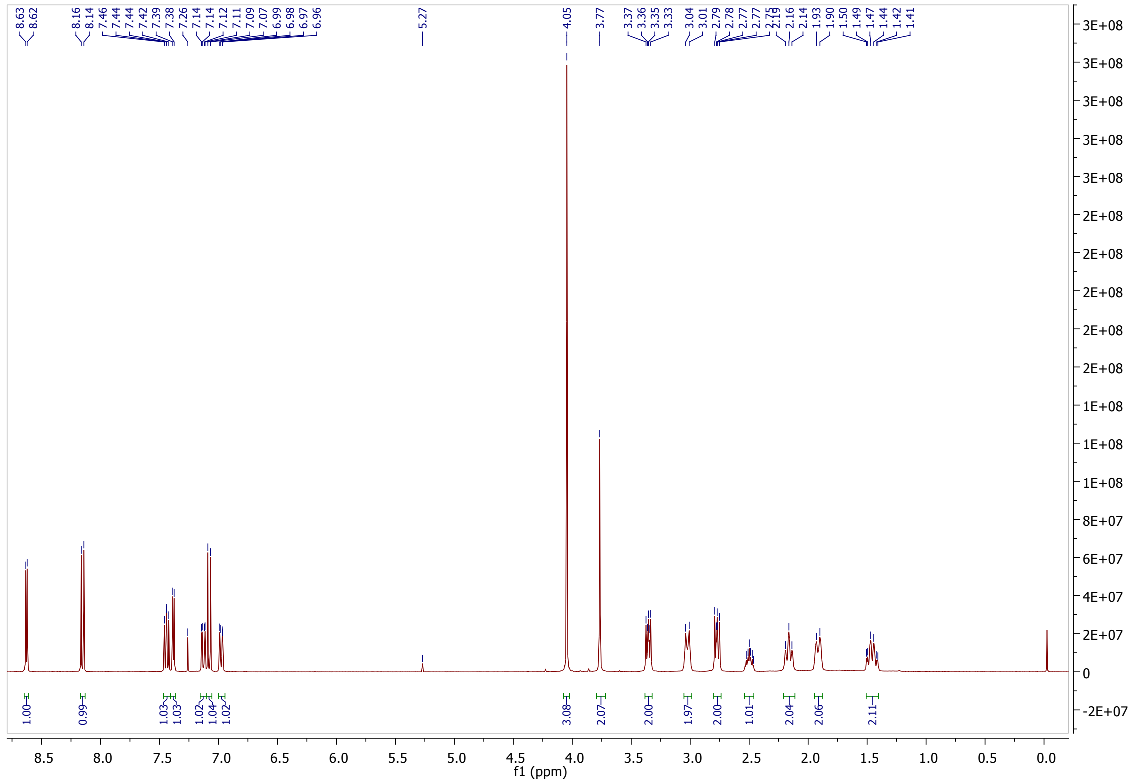

^{13}C NMR (100 MHz, $CDCl_3$)

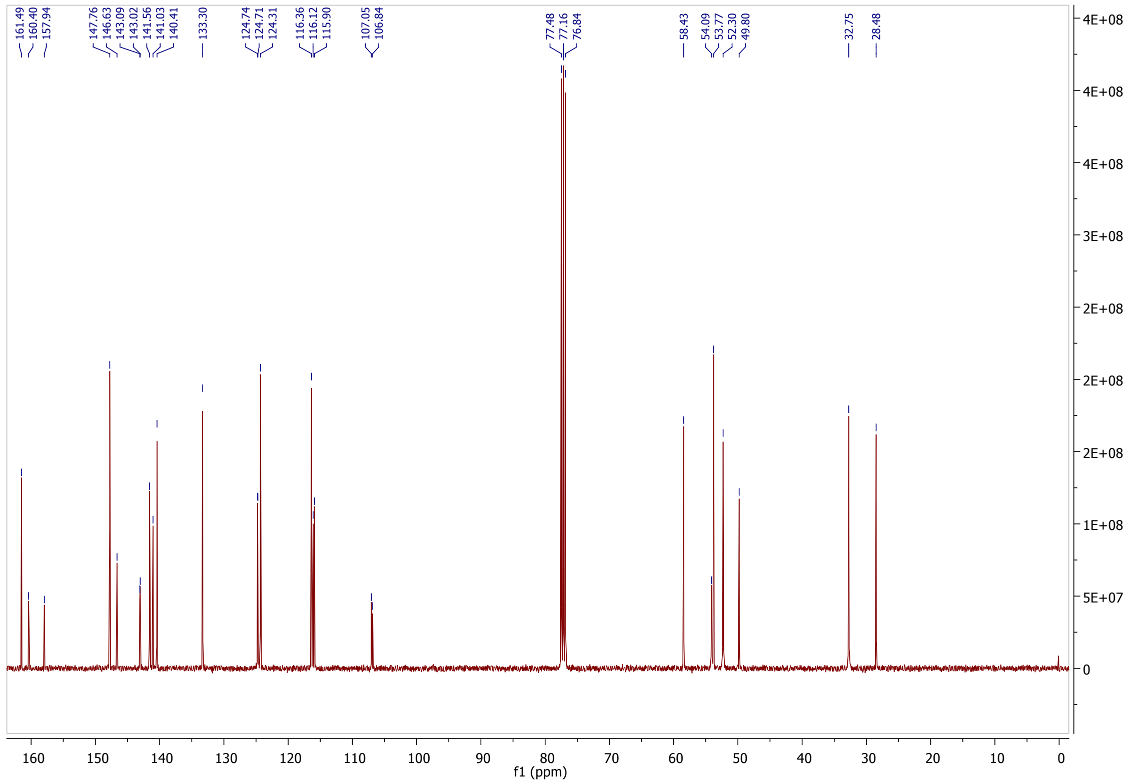

***N*-(3-fluoro-4-iodobenzyl)-1-(2-(6-methoxy-1,5-naphthyridin-4-yl)ethyl)piperidin-4-amine (6)**

^{1}H NMR (400 MHz, $CDCl_3$)

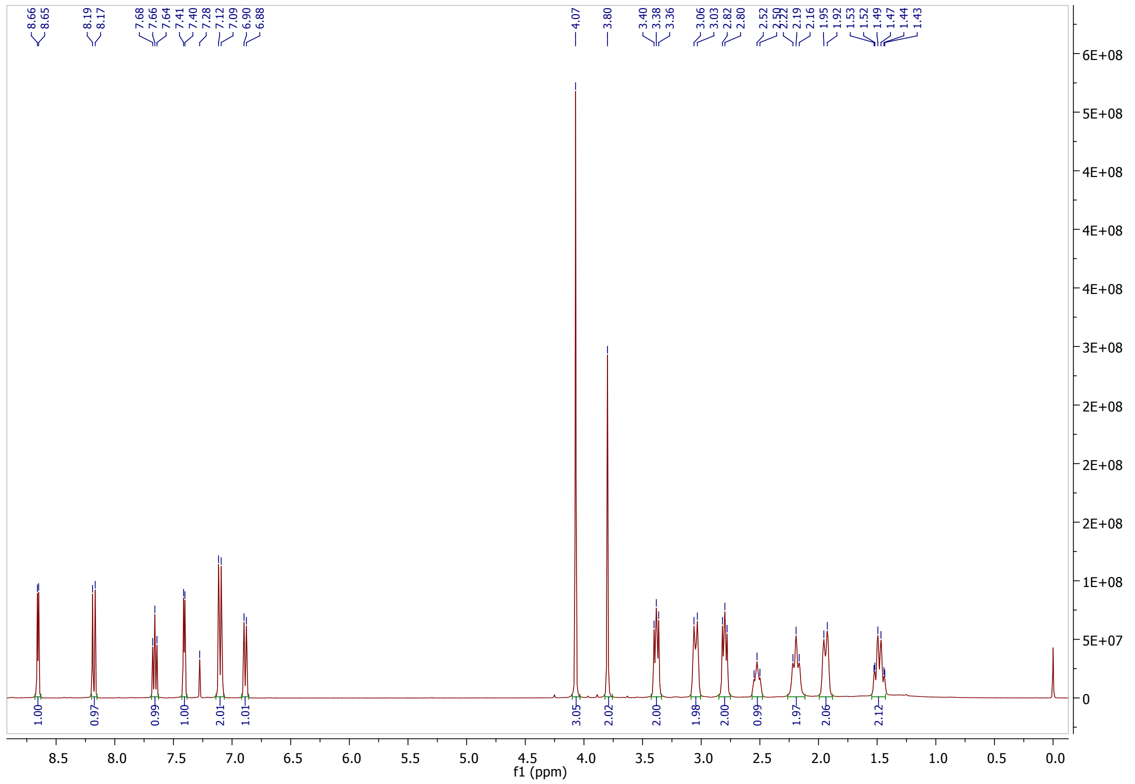

^{13}C NMR (100 MHz, $CDCl_3$)

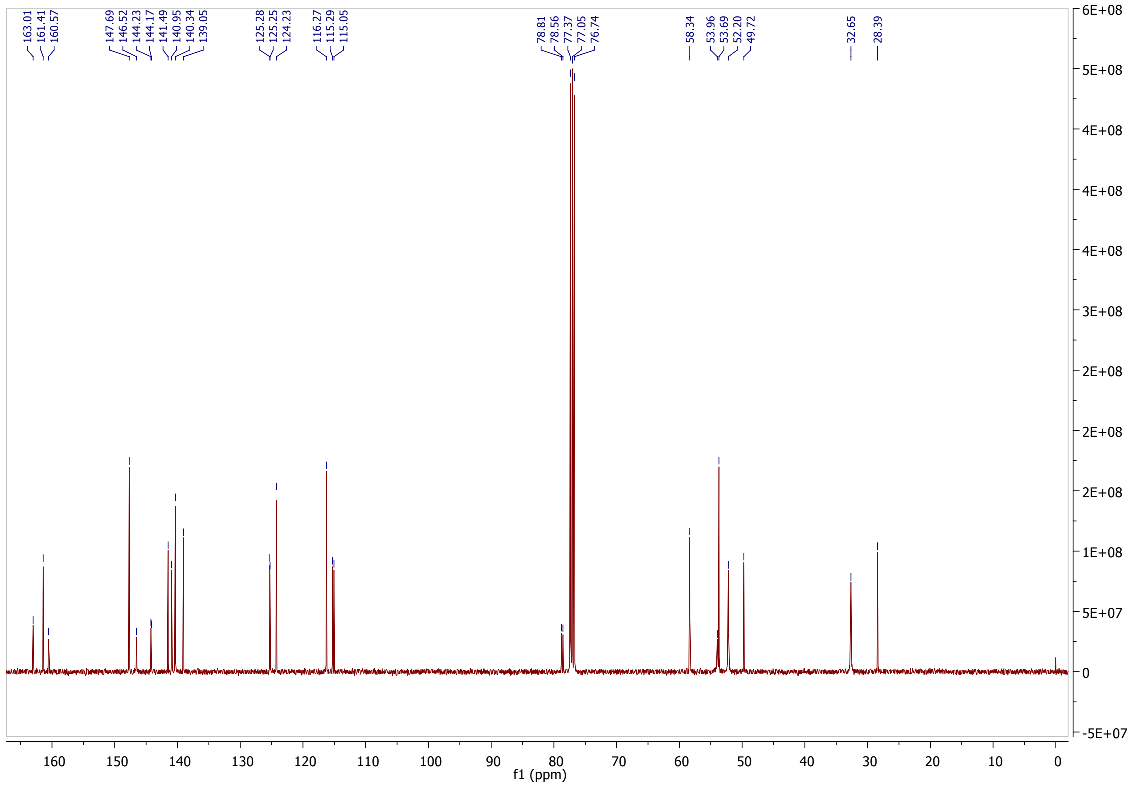

***N*-(4-chloro-3,5-difluorobenzyl)-1-(2-(6-methoxy-1,5-naphthyridin-4-yl)ethyl)piperidin-4-amine (7)**

^{1}H NMR (400 MHz, $CDCl_3$)

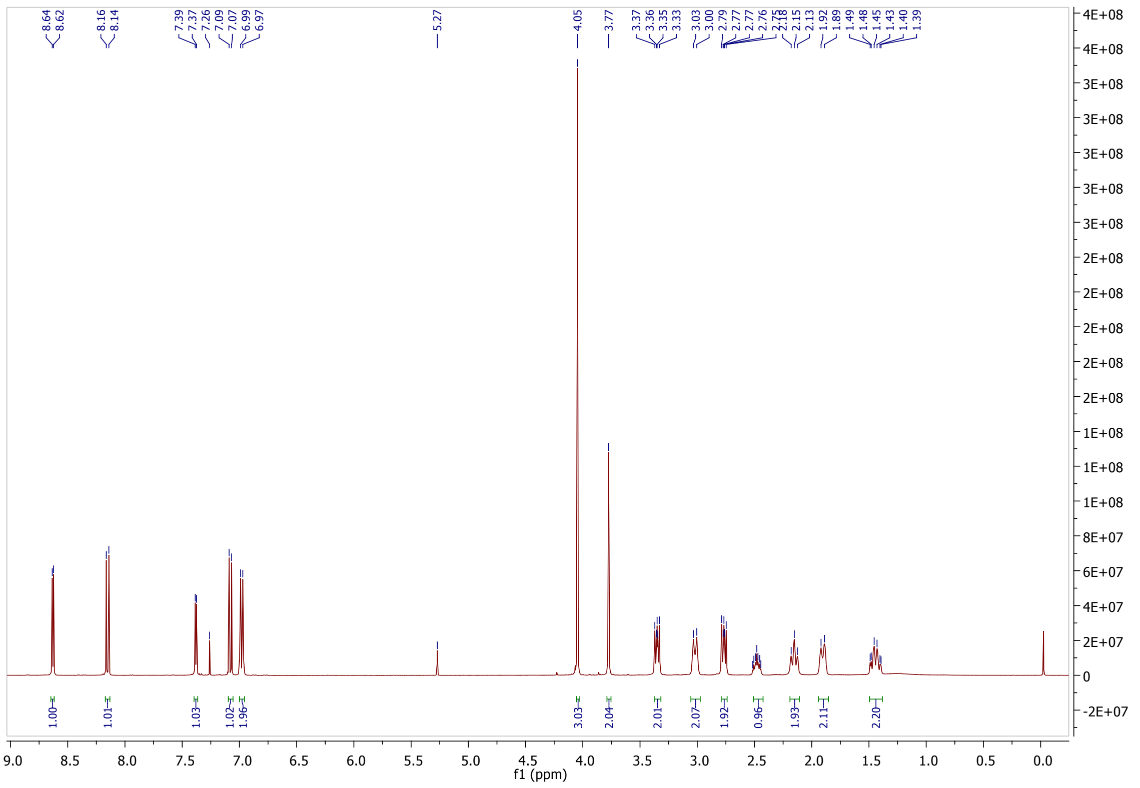

^{13}C NMR (100 MHz, $CDCl_3$)

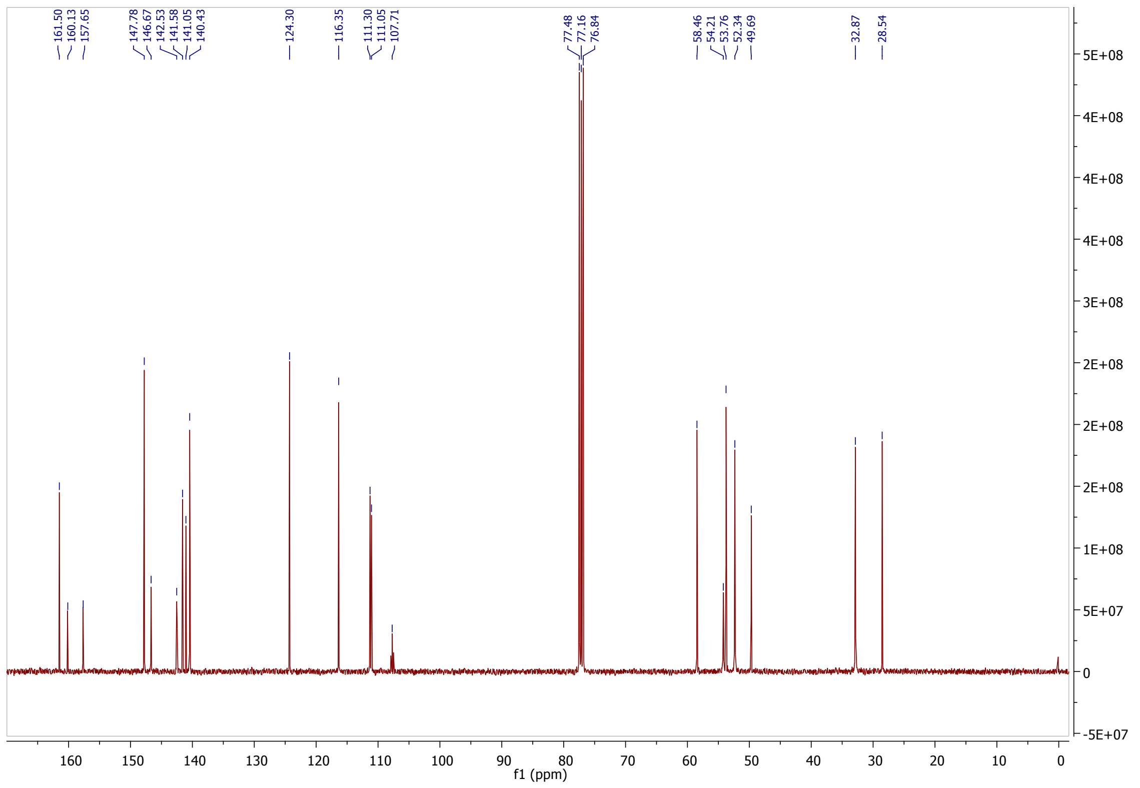

***N*-(4-bromo-3, 5-difluorobenzyl)-1-(2-(6-methoxy-1,5-naphthyridin-4-yl)ethyl)piperidin-4-amine (8)**

^{1}H NMR (400 MHz, $CDCl_3$)

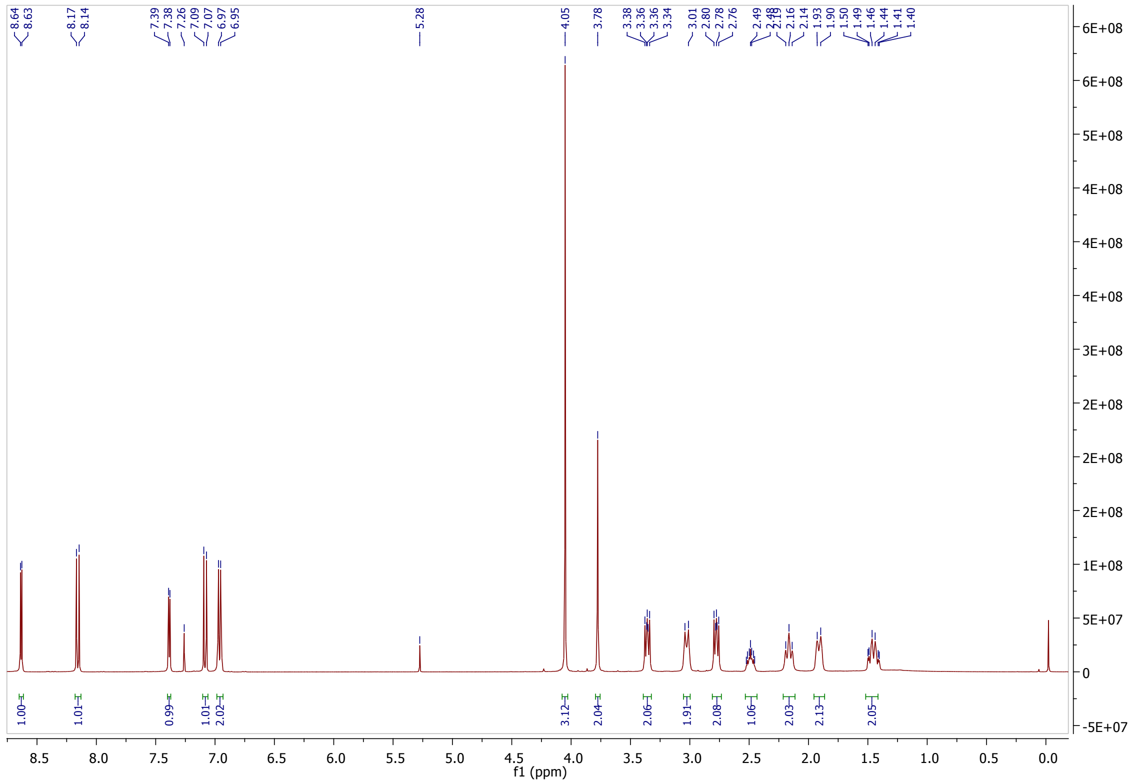

^{13}C NMR (100 MHz, $CDCl_3$)

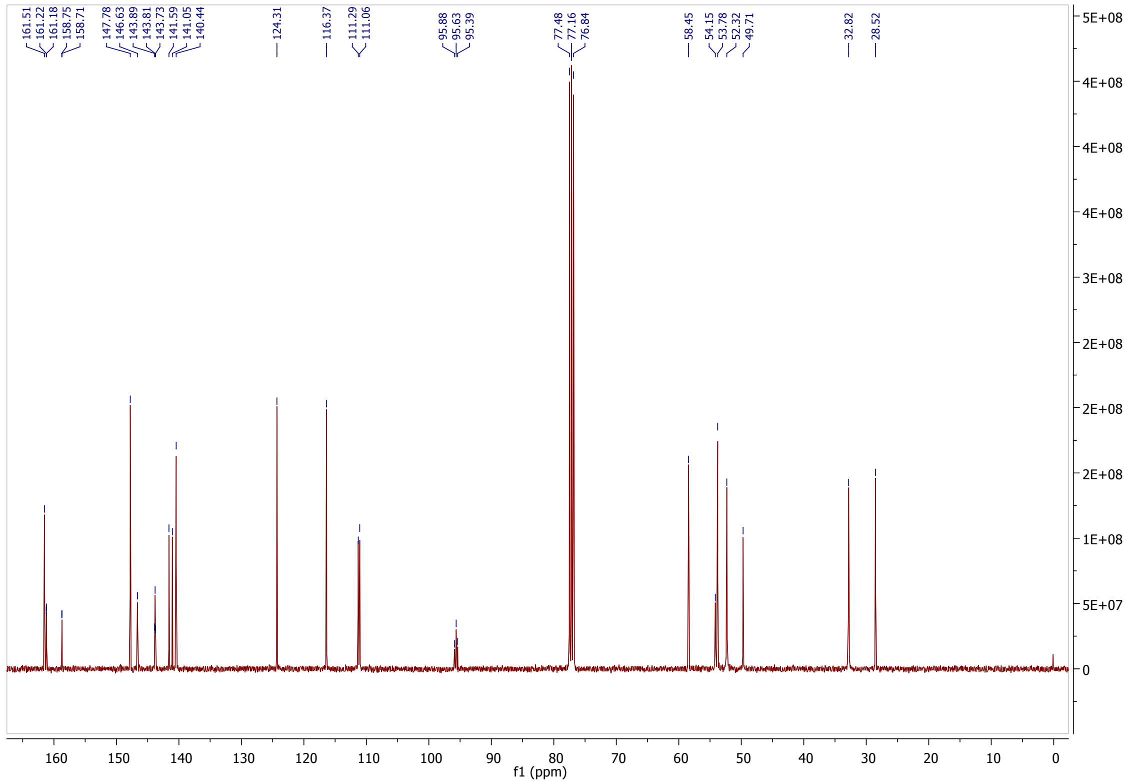

***N*-(4-bromo-2, 5-difluorobenzyl)-1-(2-(6-methoxy-1,5-naphthyridin-4-yl)ethyl)piperidin-4-amine (9)**

^{1}H NMR (400 MHz, $CDCl_3$)

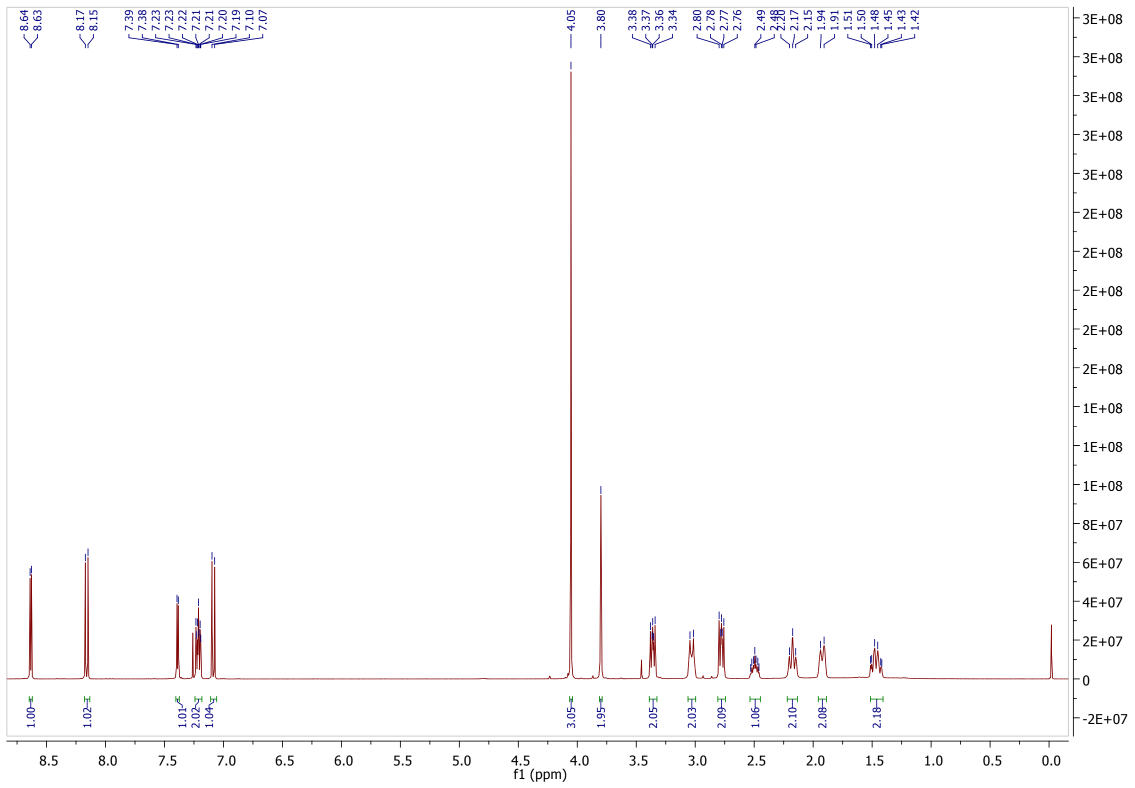

^{13}C NMR (100 MHz, $CDCl_3$)

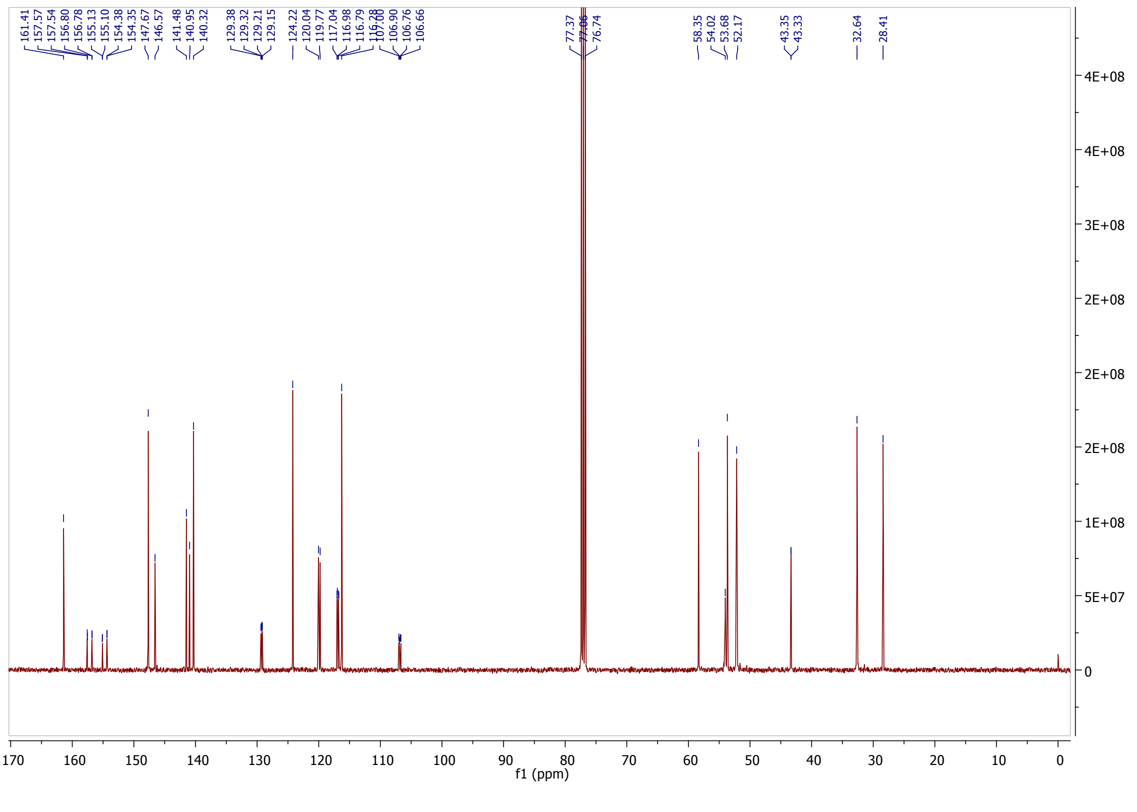

N-(4-bromo-2,3-difluorobenzyl)-1-(2-(6-methoxy-1,5-naphthyridin-4-yl)ethyl)piperidin-4-amine (10)

^{1}H NMR (400 MHz, $CDCl_3$)

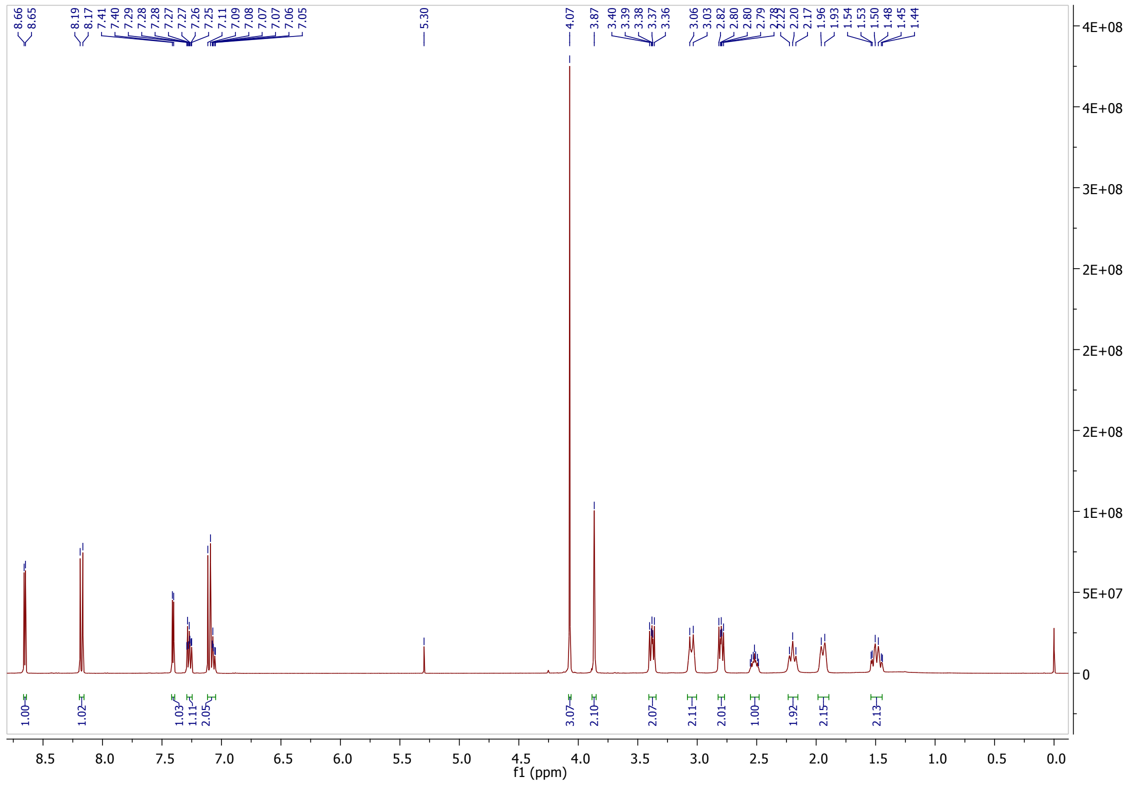

^{13}C NMR (100 MHz, $CDCl_3$)

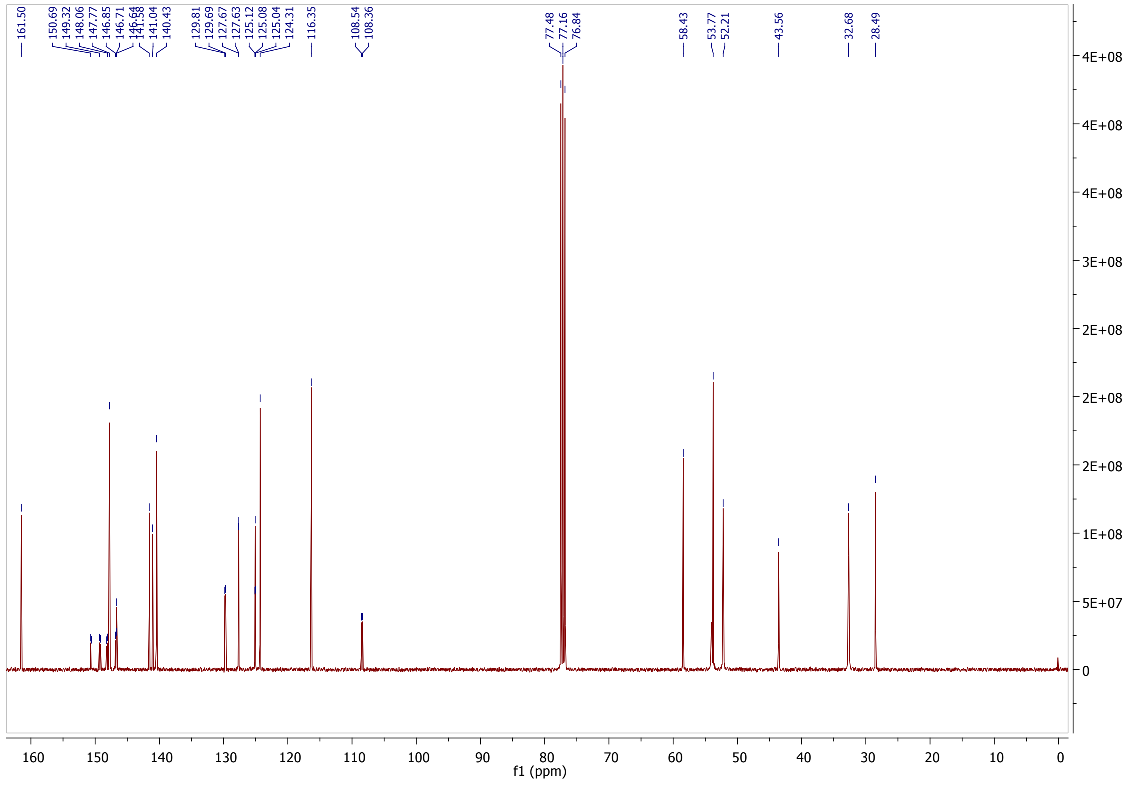

_N_-(2,3-difluoro-4-iodobenzyl)-1-(2-(6-methoxy-1,5-naphthyridin-4-yl)ethyl)piperidin-4-amine (11)

^{1}H NMR (400 MHz, $CDCl_3$)

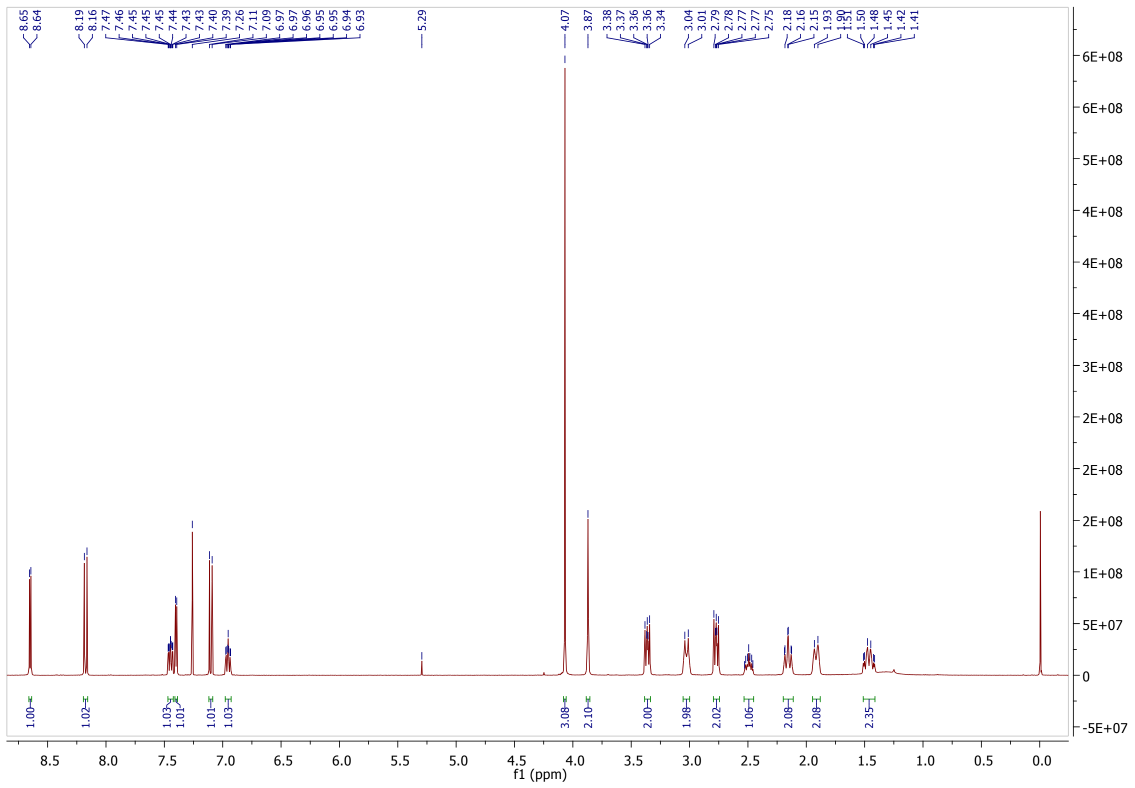

^{13}C NMR (100 MHz, $CDCl_3$)

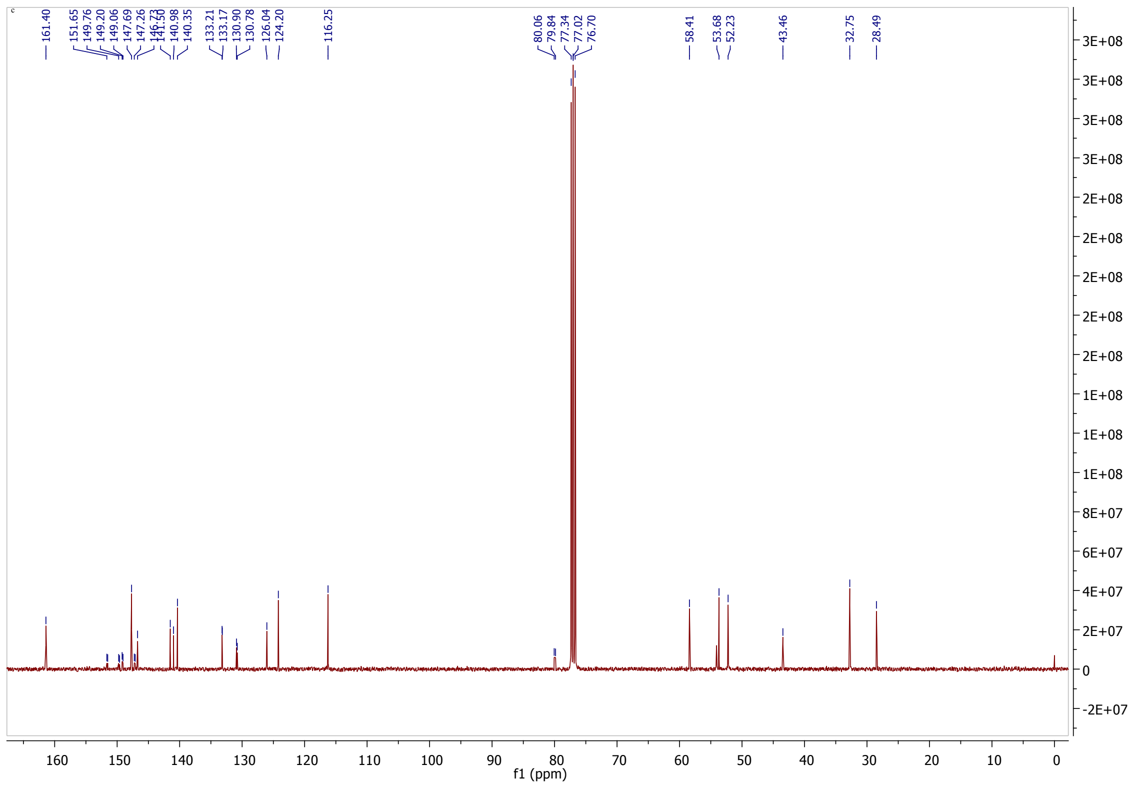

N-((6-chloropyridin-3-yl)methyl)-1-(2-(6-methoxy-1,5-naphthyridin-4-yl)ethyl)piperidin-4-amine (12)

^{1}H NMR (400 MHz, $CDCl_3$)

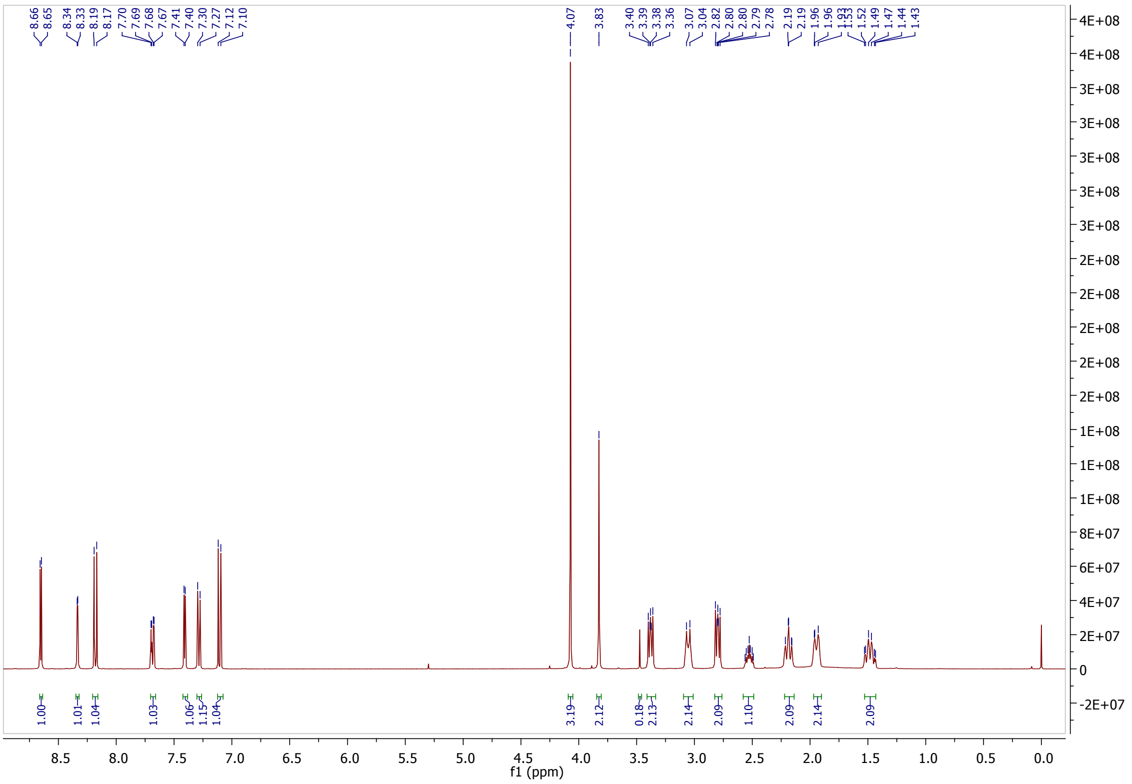

^{13}C NMR (100 MHz, $CDCl_3$)

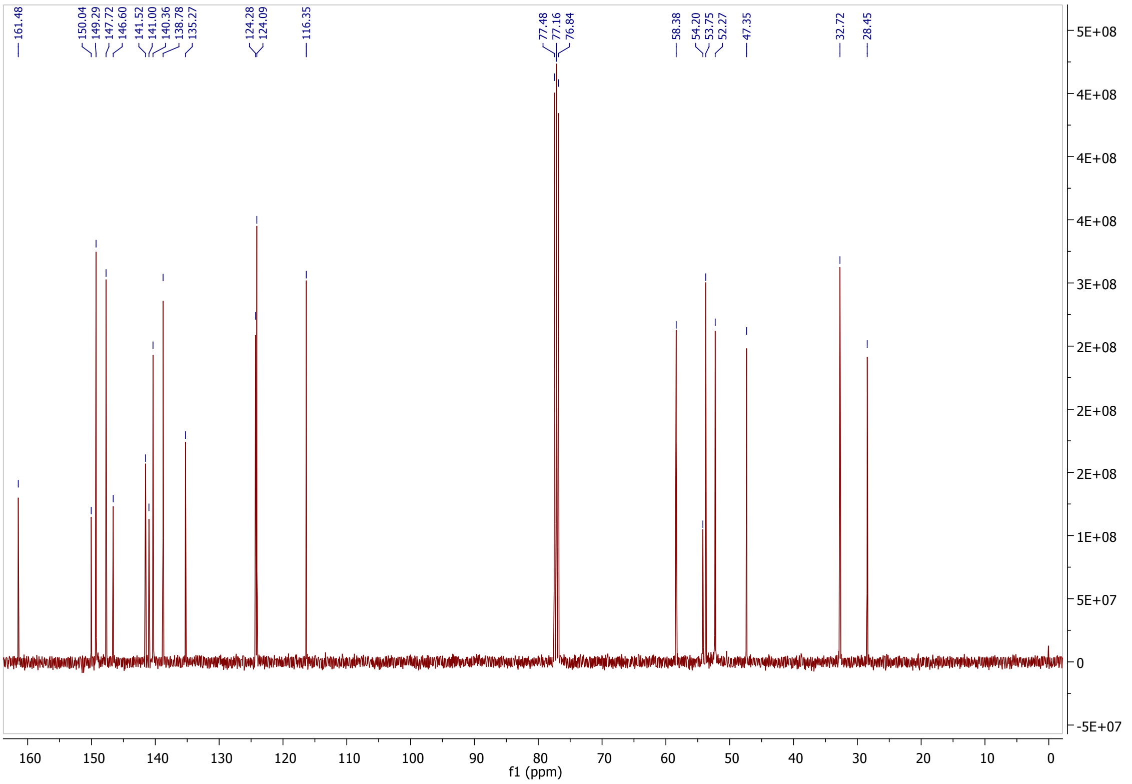

***N*-((5-chloropyridin-2-yl)methyl)-1-(2-(6-methoxy-1,5-naphthyridin-4-yl)ethyl)piperidin-4-amin (13)**

^{1}H NMR (400 MHz, $CDCl_3$)

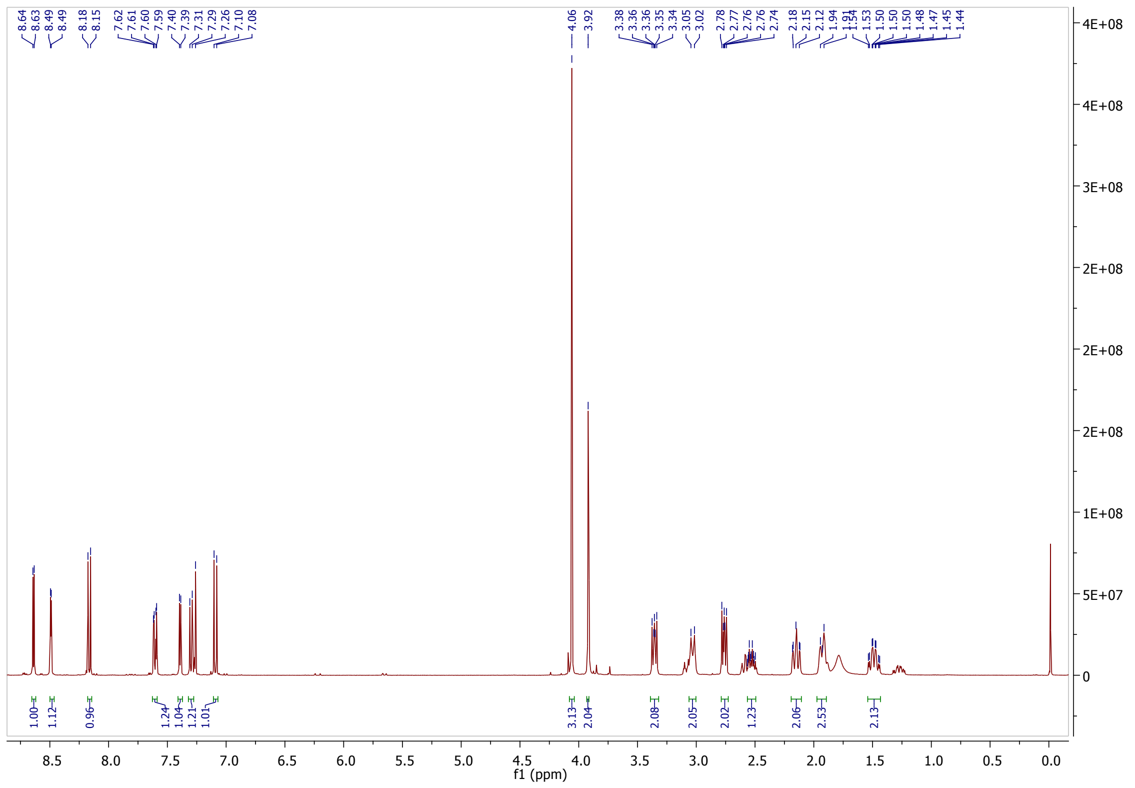

^{13}C NMR (100 MHz, $CDCl_3$)

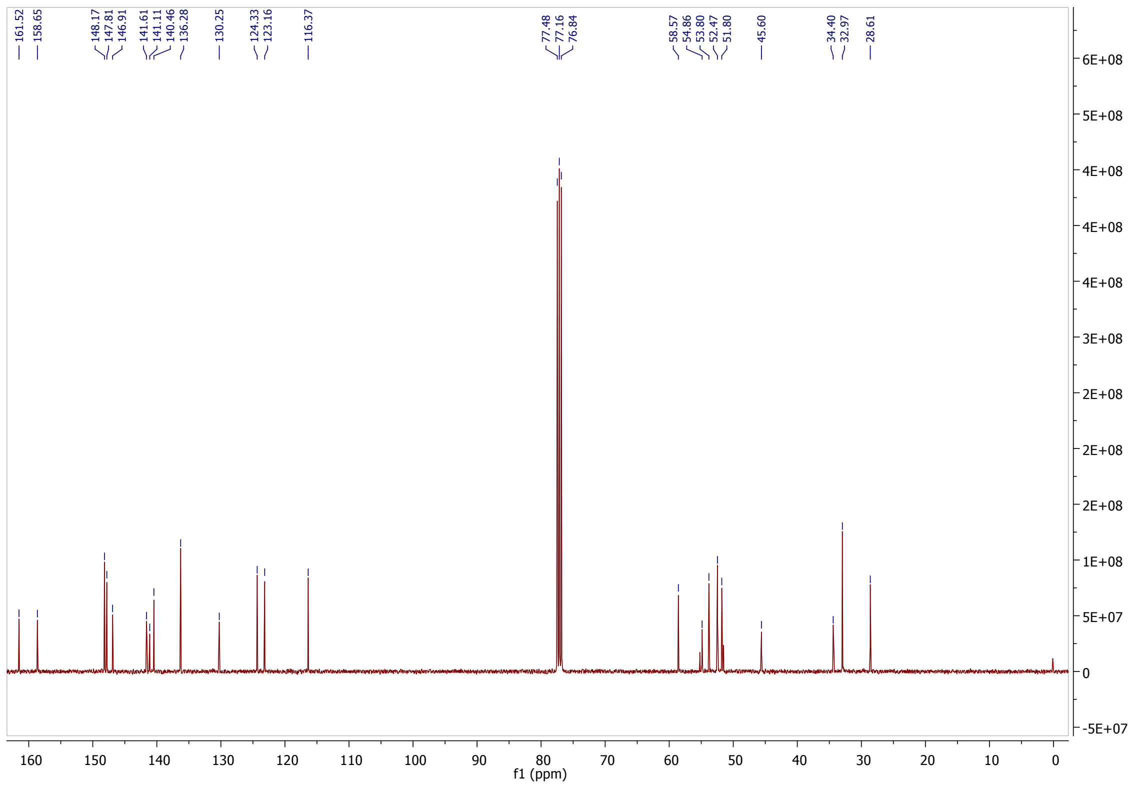

Biological evaluation

We synthesized and biologically evaluated ten new compounds. We determined their inhibitory activity, expressed as IC_{50} values, on both Gram-positive *S. aureus* and Gram-negative *E. coli* bacterial enzymes (DNA gyrase and topo IV), respectively. The *in vitro* antibacterial activity, expressed as minimum inhibitory concentration (MIC), was determined against broad spectrum of bacteria, including Gram-positive (*Staphylococcus aureus*, methicillin-resistant *Staphylococcus aureus*, *Streptococcus agalactiae*, and *Enterococcus faecalis*) and Gram-negative strains (*Escherichia coli*, *Salmonella alachua*, *Pseudomonas aeruginosa*, and *Klebsiella pneumoniae*), enriched with various MRSA strains resistant to various antibiotics as well as *E. coli* strains with increased membrane permeability and lack of efflux pump, as well. Furthermore, the selectivity of compounds for bacterial enzyme compared to the orthologous human topoisomerase IIα was also considered. The *in vitro* safety profile was determined by cytotoxicity assessment on human umbilical vein endothelial cell lines (HUVEC) and HepG2 liver cancer cell lines.

Determination of DNA gyrase and topoisomerase IV inhibitory activities

To determine the IC_{50} values of the compounds, a Gyrase Supercoiling High Throughput Plate assay kit and a TopoIV Relaxation High Throughput Plate assay kit, commercially available from Inspiralis (Norwich, UK), were used for *S. aureus* and *E. coli*. The assays were performed on black streptavidin-coated 96-well microtiter plates (Thermo Scientific Pierce, Norwich, UK) and the wells were first rehydrated with the supplied wash buffer (20 mM Tris-HCl (pH 7.6), 137 mM NaCl, 0.005% [w/v] BSA and 0.05% [v/v] Tween-20). Biotinylated oligonucleotide diluted with the wash buffer was then immobilized in each well and the excess was removed with the wash buffer. 1.5 U of the enzyme *S. aureus* or *E. coli* DNA gyrase (topoIV) was incubated together with 0.75 µg of relaxed (supercoiled) pNO1 plasmid as substrate in the presence of 3 µl inhibitor solution in 10% DMSO and 0.008% Tween-20 at 37°C for 30 min in a final reaction volume of 30 µl in the assay buffer (*S. aureus* DNA gyrase: 40 mM HEPES.KOH (pH 7.6), 10 mM magnesium acetate, 10 mM DTT, 2 mM ATP, 500 mM potassium glutamate and 0.05 mg/ml albumin; *E. coli* DNA gyrase: 35 mM Tris.HCl (pH 7.5), 24 mM KCl, 4 mM $MgCl_2$, 2 mM DTT, 1.8 mM spermidine, 1 mM ATP, 6.5 % (w/v) glycerol and 0.1 mg/ml albumin*;* *S. aureus* topo IV: 50 mM Tris.HCl (7.5), 5 mM $MgCl_2$, 5 mM DTT, 1.5 mM ATP, 350 mM potassium glutamate and 0.05 mg/ml albumin; *E. coli* topo IV: 40 mM HEPES.KOH (pH 7.6), 100 mM potassium glutamate, 10 mM magnesium acetate, 10 mM DTT, 1 mM ATP and 50 µg/ml albumin). The reaction was stopped by adding the TF buffer (50 mM NaOAc (pH 4.7), 50 mM NaCl and 50 mM $MgCl_2$) to allow triplex formation (biotin oligonucleotide plasmid) for another 30 min. Finally, the unbound plasmid was washed off using the TF buffer and the Promega Diamond dye was added to T10 buffer (10 mM Tris-HCl (pH 8.0), 1 mM EDTA) solution. After 15 min the solution was mixed and the fluorescence was read off with a Tecan Fluorimeter (excitation, 495 nm; emission, 537 nm). Two inhibitor concentrations of 100 and 1 µM were used for pre-screening. The IC_{50} values were determined at seven inhibitor concentrations for those compounds that showed a residual enzyme activity of less than 50% at the concentration 100 µM, while the other compounds were noted as inactive (IC_{50} >100 µM). The inhibitor concentration at which the residual enzyme activity is 50% (IC_{50}) was calculated by nonlinear regression-based fitting of the inhibition curves using the log [inhibitor] versus response-variable slope (four parameters) – symmetrical equation, in GraphPad Prism 6.0 software (GraphPad Software, CA, USA). The IC_{50} value represents the average of two independent measurements. Gepotidacin was used as a positive control, showing IC_{50} for DNA gyrase 0.374 µM and 0.244 µM for *S. aureus* and *E. coli* and topo IV 8.30 µM and 0.049 µM for *S. aureus* and *E. coli,* respectively.

Antimicrobial testing

The minimum inhibitory concentrations (MICs) were determined using the broth micro-dilution method in 96-well plate format according to the Clinical and Laboratory Standards Institute

guidelines[8] and European Committee on Antimicrobial Susceptibility Testing recommendations[9]. The bacterial suspension of the specific bacterial strain equivalent to the 0.5 McFarland turbidity standard was diluted with cation-adapted Mueller Hinton broth with TES (Thermo Fisher Scientific) to obtain a final inoculum of 10^5 CFU/ml. The compounds dissolved in DMSO and inoculum were mixed together and incubated for 20 h at 35 °C. After incubation the MIC values were determined by visual inspection, as the lowest dilution of the compounds showed no turbidity. MICs were determined against various bacterial strains. Tetracycline was used as a positive control on each assay plate.

Table S3. Antimicrobial susceptibility of the optimized NBTIs against a panel of various Gram-positive and Gram-negative bacterial pathogens.

Cmpd	MIC [µM]													
	**1***	**2***	**3***	**4**	**5**	**6**	**7**	**8**	**9**	**10**	**11**	**12**	**13**	**Gepo**
S. aureus (ATCC 29213)	0.304	0.068	0.016	0.072	0.017	0.015	0.018	0.008	0.016	0.063	0.015	2.43	0.303	0.279
E. coli (ATCC 25922)	9.73	4.39	3.98	4.66	2.11	0.961	1.12	1.02	2.04	8.14	3.71	77.7	77.7	2.23
E. coli D2 [a]	4.87	0.274	0.249	0.583	0.131	0.119	0.069	0.033	0.509	1.018	0.23	9.71	38.84	0.279
E. coli N43[b] (CGSC# 5583)	1.22	0.274	0.155	0.145	0.065	0.031	0.139	0.016	0.126	0.254	0.058	19.4	19.4	0.036
**MRSA** (QA-11.7)[c]	1.22	0.136	0.016	0.072	0.034	0.031	0.069	0.033	0.033	0.126	0.058	2.43	0.607	0.138
**MRSA** (QA-12.1)[d]	ND	ND	ND	0.072	0.065	0.031	0.069	0.033	0.033	0.126	0.115	4.86	0.607	0.279
K. pneumoniae	ND	ND	ND	74.6	33.8	15.4	17.9	16.3	130	130	59.4	>311	>311	17.8
S. alachua RDK 030c (QA-1482/04)	77.9	35.1	15.9	18.7	8.45	3.84	4.47	2.04	32.6	32.6	14.9	>311	>311	8.92
P. aeruginosa RDK 184 (DSM 939)	>311	281	63.7	149	67.6	30.7	35.8	16.3	260	>260	119	>311	>311	17.8
S. agalactiae RDK 047 (QA-990/02)	2.43	1.098	0.249	2.33	4.22	0.480	1.12	0.509	0.509	4.07	0.929	155	19.4	ND
E. faecalis DRK 057 (ATCC 29212)	2.43	2.20	1.99	4.66	1.06	0.480	1.12	1.02	1.02	4.07	0.929	77.7	9.71	ND

[a] Bears a mutation in the lpxC gene that increases membrane permeability. [b] AcrA knockout strain (knockout of cell membrane efflux pump). [c] Resistant to: cefoxitin, ciprofloxacin, clindamycin, erythromycin, tetracycline, thiamulin, trimethoprim. [d] Resistant to: cefoxitin, gentamicin, kanamycin, rifampicin, streptomycin, sulfamethoxazole, tetracycline; Gepo: gepotidacin; ND: not determined; *reference[10].

Cytotoxycity (Metabolic activity assay)

HepG2 (ATCC) and HUVEC (ATCC) cell lines were cultured in Dulbecco's Modified Eagle Medium (DMEM; Sigma-Aldrich, St. Louis, MO, USA) supplemented with 10% fetal bovine serum (Gibco, Grand Island/NY, USA), 2 mM L-glutamine, 100 U/mL penicillin, 100 µg/mL streptomycin (all from Sigma-Aldrich) in a humidified chamber at 37 °C and 5% CO_2. Cells were seeded into 96-well plates at a density of 5,000 cells per well (HUVEC) or 8,000 cells per well (HepG2) and allowed to attach overnight. After 24 h cells were treated with compound of interest or corresponding vehicle as

control. The metabolic activity was assessed after 72 h treatment using the CellTiter96 Aqueous One Solution Cell Proliferation Assay (Promega, Madison/WI, USA). The absorbance was measured at 492 nm on an automated microplate reader Synergy™ 4 Hybrid Microplate Reader (BioTek, Winooski, VT, USA). The data were normalized and IC_{50} were calculated with GraphPad prism 8.2.1 software using a nonlinear regression. Results are presented as IC_{50} values of each compound from three independent experiments, each conducted in duplicate. Results for **12** and **13** are presented as %RA values of each compound from two independent experiments, each conducted in triplicate.

Correlation of inhibitory and antibacterial activity

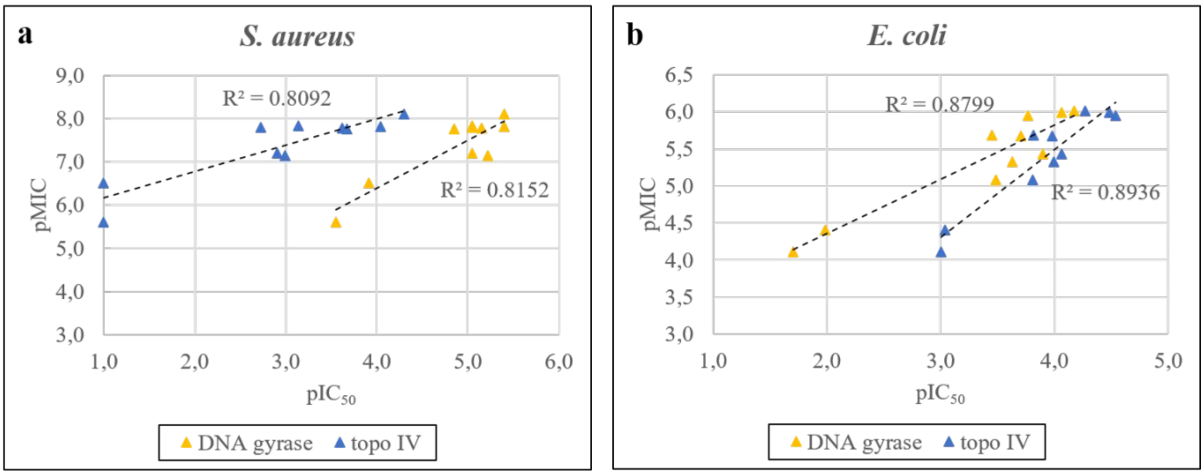

Figure S5: The correlation between antibacterial potency (pMIC = -logMIC in molar) of NBTIs and the corresponding DNA gyrase and topo IV inhibitory activity (pIC_{50} = -logIC_{50} in molar) of: **a)** *S. aureus*, and **b)** *E. coli*, respectively. A high correlation may be observed for *E. coli* for both enzymes (R^2 = 0.8799 and 0.8936 for DNA gyrase and topo IV, respectively), while slightly inferior linear correlation can be obtained for *S. aureus* (R^2 = 0.8152 and 0.8092 for DNA gyrase and topo IV).

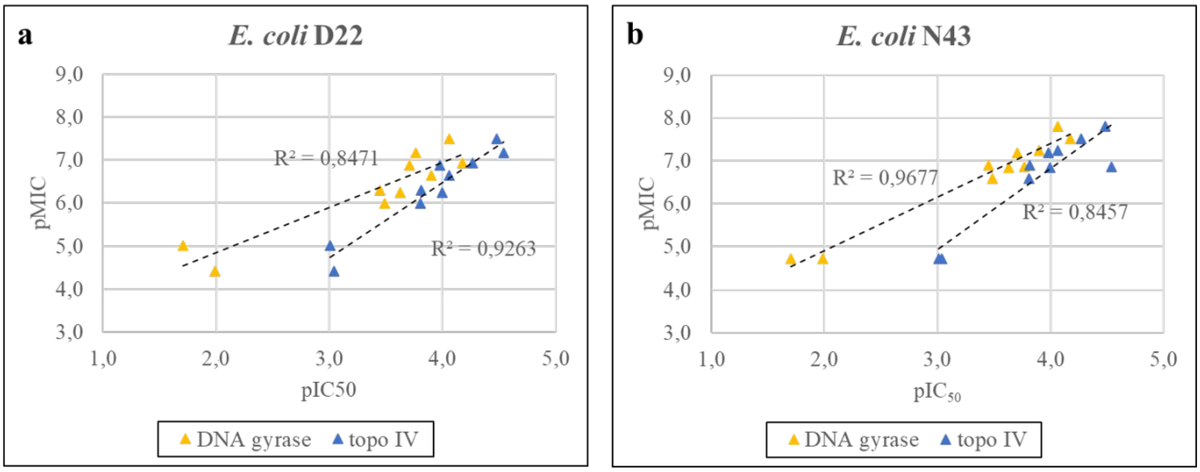

Figure S6: The correlation between antibacterial potency (pMIC = -logMIC in molar) of NBTIs and the corresponding DNA gyrase and topo IV inhibitory activity (pIC_{50} = -logIC_{50} in molar) of: **a)** *E. coli* D22 strain and **b)** *E. coli* N43 strain (with removed efflux pumps), respectively. A significantly higher correlation may be observed for *E. coli* N43 for both enzymes (R^2 = 0.9677 and 0.8457 for DNA gyrase and topo IV, respectively), while in case of *E. coli* D22 no correlation was observed (R^2 = 0.8471 and 0.9263 for DNA gyrase and topo IV).

References

(1) Waterhouse, A.; Bertoni, M.; Bienert, S.; Studer, G.; Tauriello, G.; Gumienny, R.; Heer, F. T.; De Beer, T. A. P.; Rempfer, C.; Bordoli, L.; Lepore, R.; Schwede, T. SWISS-MODEL: Homology Modelling of Protein Structures and Complexes. *Nucleic Acids Res.* **2018**, *46* (W1), W296–W303.

(2) https://www.rcsb.org/structure/3RAF.

(3) Laponogov, I.; Pan, X. S.; Veselkov, D. A.; Mcauley, K. E.; Fisher, L. M.; Sanderson, M. R. Structural Basis of Gate-DNA Breakage and Resealing by Type II Topoisomerases. *PLoS One* **2010**, *5* (6).

(4) Van Zanten, A. Development and Validation of a Genetic Algorithm for Flexible Docking Gareth. *Form. Empl.* **2012**, *120* (4), 127–134.

(5) Kolarič, A.; Germe, T.; Hrast, M.; Stevenson, C. E. M.; Lawson, D. M.; Burton, N. P.; Vörös, J.; Maxwell, A.; Minovski, N.; Anderluh, M. Potent DNA Gyrase Inhibitors Bind Asymmetrically to Their Target Using Symmetrical Bifurcated Halogen Bonds. *Nat. Commun.* **2021**, *12* (1), 1–13.

(6) Vanden Broeck, A.; Lotz, C.; Ortiz, J.; Lamour, V. Cryo-EM Structure of the Complete E. Coli DNA Gyrase Nucleoprotein Complex. *Nat. Commun.* **2019**, *10* (1), 1–12.

(7) Bax, B. D.; Chan, P. F.; Eggleston, D. S.; Fosberry, A.; Gentry, D. R.; Gorrec, F.; Giordano, I.; Hann, M. M.; Hennessy, A.; Hibbs, M.; Huang, J.; Jones, E.; Jones, J.; Brown, K. K.; Lewis, C. J.; May, E. W.; Saunders, M. R.; Singh, O.; Spitzfaden, C. E.; Shen, C.; Shillings, A.; Theobald, A. J.; Wohlkonig, A.; Pearson, N. D.; Gwynn, M. N. Type IIA Topoisomerase Inhibition by a New Class of Antibacterial Agents. *Nature* **2010**, *466* (7309), 935–940.

(8) Clinical Laboratory Standards Institute. Methods for Dilution Antimicrobial Susceptibility Tests for Bacteria That Grow Aerobically ; Approved Standard — Ninth Edition. CLSI Document M07-A9. *Clin. Lab. Standars Inst.* **2018**, *32* (2), 18.

(9) EUCAST. Testing Breakpoint Tables for Interpretation of MICs and Zone Diameters. *Https://Www.Eucast.Org/Ast_of_Bacteria/* **2020**, 0–77.

(10) Kolarič, A.; Kokot, M.; Hrast, M.; Weiss, M.; Zdovc, I.; Trontelj, J.; Žakelj, S.; Anderluh, M.; Minovski, N. A Fine-Tuned Lipophilicity / Hydrophilicity Ratio Governs Antibacterial Potency and Selectivity of Bifurcated Halogen. *Antibiotics.* **2021**, *10*, 862.
